# N-doped carbon dots for dual-modality NIR fluorescence imaging and photothermal therapy

**DOI:** 10.1186/s12951-025-03497-6

**Published:** 2025-07-15

**Authors:** Hui-Xian Shi, Xuan Qu, Tong-Tong Zhao, Zhong-Fu An, Chuan-Yi Zhang, Hong-Liang Wang

**Affiliations:** 1 Shanxi Key Laboratory of Biomedical Metal Materials, College of Materials Science and Engineering, Taiyuan University of Technology, Taiyuan, 030024 China; 2 Key Laboratory of Flexible Electronics (KLOFE) & Institute of Advanced Materials (IAM), Nanjing Tech University, 30 South Puzhu Road, Nanjing, 211816 China; 3 Department of Nuclear Medicine, First Hospital of Shanxi Medical University, Taiyuan, Shanxi 030001 China; 4 Shanxi Key Laboratory of Molecular Imaging & Collaborative Innovation Center for Molecular Imaging of Precision Medicine, Shanxi Medical University, Taiyuan, 030001 Shanxi China

**Keywords:** Carbon Dots, Near-infrared II window, Bioluminescence imaging, Photothermal therapy, Tumor elimination

## Abstract

Photothermal therapy (PTT), a rapidly advancing non-invasive cancer treatment modality, utilizes photothermal agents to convert light energy into thermal energy, enabling precise and localized destruction of cancer cells. Recent developments have focused on photothermal agents operating in the second near-infrared (NIR-II) biological window (1000–1350 nm), which offer enhanced tissue penetration depth and improved therapeutic precision for deep-seated tumors while minimizing collateral damage to healthy tissues. In this study, we developed a novel class of nitrogen-doped carbon dots (N-CDs) through a facile one-pot hydrothermal synthesis approach. The synthesized N-CDs demonstrate remarkable dual functionality, exhibiting both superior photothermal performance and fluorescence imaging capabilities within the NIR region, thereby enabling simultaneous tumor diagnosis and therapy. These N-CDs display exceptional biocompatibility and achieve impressive photothermal conversion efficiencies of 31.25% and 27.12% under 808 nm and 1060 nm laser irradiation, respectively, with corresponding temperature changes of 42.8 ℃ and 39.7 ℃ in vitro. Notably, the N-CDs exhibit a strong fluorescence emission peak at 660 nm, approaching the NIR-I window, which facilitates high-contrast bioimaging. In vivo studies confirmed the therapeutic efficacy of N-CDs, demonstrating cancer cell ablation under both 808 nm and 1060 nm laser irradiation, coupled with accurate tumor localization capabilities. The unique combination of intense fluorescence emission, exceptional photothermal conversion efficiency, and outstanding biocompatibility positions these N-CDs as a highly promising theranostic platform for integrated cancer diagnosis and treatment.

**Supplementary Information:**

The online version contains supplementary material available at 10.1186/s12951-025-03497-6.

## Graphical Abstract

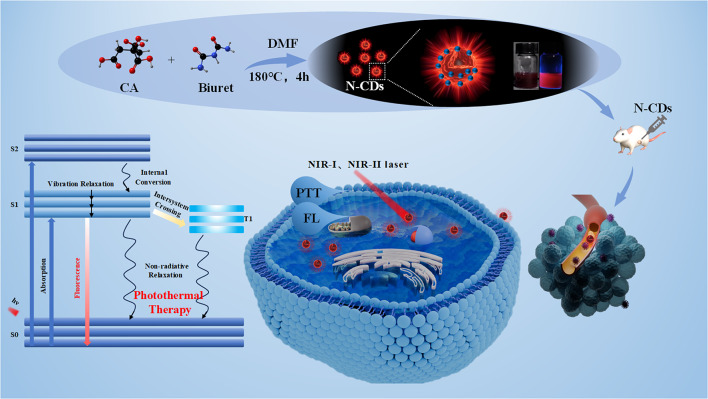

## Introduction

In recent years, the global burden of malignant tumors has significantly increased, with both incidence and mortality rates showing a steady upward trend, making cancer one of the most pressing public health challenges worldwide [1]. Conventional cancer treatment modalities, including radiotherapy and chemotherapy, often lead to substantial collateral damage to healthy tissues and systemic toxicity. In contrast, emerging therapeutic strategies such as photothermal therapy (PTT) combined with fluorescence imaging have emerged as promising alternatives. These innovative approaches offer non-invasive, non-ionizing treatment options that minimize damage to surrounding healthy tissues while enabling precise, targeted therapy with reduced systemic toxicity [2, 3]. PTT operates through the utilization of near-infrared (NIR) absorbing agents that convert laser energy into localized thermal energy, selectively ablating cancer cells while sparing normal tissues. Compared to conventional chemotherapy, PTT provides distinct advantages including precise spatial control, enhanced therapeutic specificity, and minimal invasiveness. To achieve optimal therapeutic outcomes, an ideal PTT agent should possess three key characteristics: strong NIR absorption capacity, high photothermal conversion efficiency, and excellent biocompatibility with minimal systemic toxicity [4, 5].

One of the primary limitations in conventional PTT is the restricted tissue penetration depth of first near-infrared (NIR-I, 650–950 nm) light, which often results in suboptimal therapeutic outcomes, particularly foe deep-seated tumors [6]. In contrast, second near-infrared (NIR-II, 1000–1350 nm) window offers significant advantages, including enhanced tissue penetration depth, higher maximum permissible laser exposure thresholds, improved signal-to-background ratio, and reduced tissue scattering [7, 8]. These superior optical properties make NIR-II light particularly promising for advancing PTT applications. Over the past decade, a wide range of photothermal agents has been investigated, including noble metal nanomaterials (e.g., Au, Pt, and Pd), metal chalcogenides, carbon-based materials (e.g., graphene and its derivatives), two-dimensional (2D) materials (e.g., MXenes, carbon nitride, and black phosphorus), organic small molecules, and semiconducting polymer nanoparticles [9]. Despite these advancements, the development of NIR-II photothermal agents, spanning both inorganic and organic materials, still faces several critical challenges that hinder their clinical translation [10, 11].

Current NIR-II photothermal agents often suffer from limitations sch as low photothermal conversion efficiency and insufficient absorption, necessitating the use of higher laser power densities to achieve therapeutic effects [12]. Additionally, many of these agents exhibit poor biocompatibility, including elevated physiological toxicity and insufficient solubility in biological media. For example, while gold nanoparticles demonstrate exceptional photothermal conversion efficiency (> 80%) at 1064 nm, their clinical application is restricted by potential toxicity concerns and poor solubility in physiological environments [13]. Organic photothermal agents, although generally exhibiting better biocompatibility and biosafety, are often plagued by issues such as poor photostability, complex synthesis protocols, and high production costs [14]. Another significant challenge lies in ensuring the long-term biosafety of nanoscale photothermal agents. Studies have shown that nanoparticles with diameters below 5–7 nm can be efficiently cleared through renal excretion, thereby reducing the risk of long-term toxicity [15, 16]. However, many NIR-II photothermal agents exceed 50 nm in size, which impedes their renal clearance and raises concerns about their long-term biocompatibility and potential accumulation in vivo.

Furthermore, in vivo bioluminescence imaging, a highly sensitive technology, is critical in areas such as tumor metastasis research, drug development, gene therapy, and stem cell tracing [17]. This advanced imaging modality offers exceptional sensitivity, enabling the detection of minute tumor lesions comprising as few as several hundred cells. While traditional imaging methods have made significant strides, recent innovations have integrated fluorescence imaging with photothermal therapy (PTT) using multifunctional nanomaterials. For instance, laser-synthesized nanodiamonds have been employed as fluorescent cores, combining fluorescence imaging capabilities with PTT functionality [18]. In another approach, Santu et al. developed a nanospheroidal precipitation method to synthesize variable molecular weight nanoparticles (VMWNPs) incorporating oligomers and high molecular weight fragments of PCPDTBSe. These nanoparticles demonstrated dual functionality, enabling near-infrared fluorescence imaging (800 nm) and photothermal-responsive ablation of breast cancer cells [19]. Similarly, Huang and colleagues designed near-infrared fluorescent nanoparticles based on pyrrole cyanine (PPCys), which exhibit strong absorption in the NIR window (808 nm). These nanoparticles served as effective theranostic agents, demonstrating robust NIR fluorescence and photothermal effects in both in vitro and in vivo settings, thereby facilitating simultaneous cancer imaging and treatment [20]. Despite these advancements, most existing materials still face limitations, including insufficient NIR-II absorption and larger particle sizes, which compromise their biocompatibility and overall efficacy in integrated imaging and therapeutic applications.

Notably. carbon dots (CDs), a class of zero-dimensional-carbon nanoparticles with a diameter less than 10 nm, composed of a carbon core and surface functional groups [21, 22]. Owing to their nanoscale dimensions and excellent biocompatibility, CDs have found extensive use in diverse fields such as biosensing, biomedical imaging, drug delivery, and photothermal therapy (PTT) [23–25]. Recent advancements in CDs synthesis have yielded materials with remarkable photothermal conversion efficiencies. For instance, Zhao et al. and Geng et al. independently developed CDs with photothermal conversion efficiencies of 54.7% at 808 nm and 46% at 671 nm, respectively [26, 27]. Furthermore, CDs derived from hydrophobic cyanine dyes have demonstrated preferential tumor accumulation and high photothermal conversion efficiency under 808 nm laser irradiation [28].

The integration of PTT with fluorescence imaging for tumor theranostics has also been explored. Guo and colleagues synthesized Cu@CDs to enhance NIR-II absorption, demonstrating significant cancer inhibition through synergistic photothermal/photodynamic therapy. These Cu@CDs also served as dual-mode imaging agents, enabling fluorescence cell imaging and NIR thermal imaging to monitor the treatment process both in vitro and in vivo [29]. In another notable study, Ge’s group developed R-CDs for multimodal applications, including fluorescence imaging, photoacoustic diagnosis, and PTT, achieving a photothermal conversion efficiency of 38.5% under 671 nm laser irradiation [30]. Despite these promising developments, current CD-based systems face several limitations, including absorption and emission at relatively short wavelengths, potential physiological toxicity associated with transition metal components, and insufficient NIR-II absorption. These challenges highlight the critical need for developing advanced CDs with optimized properties, including strong NIR-II absorption, red-shifted fluorescence emission, and minimal physiological toxicity, to enable more effective and safer tumor diagnosis and treatment.

**Fig. 1:**
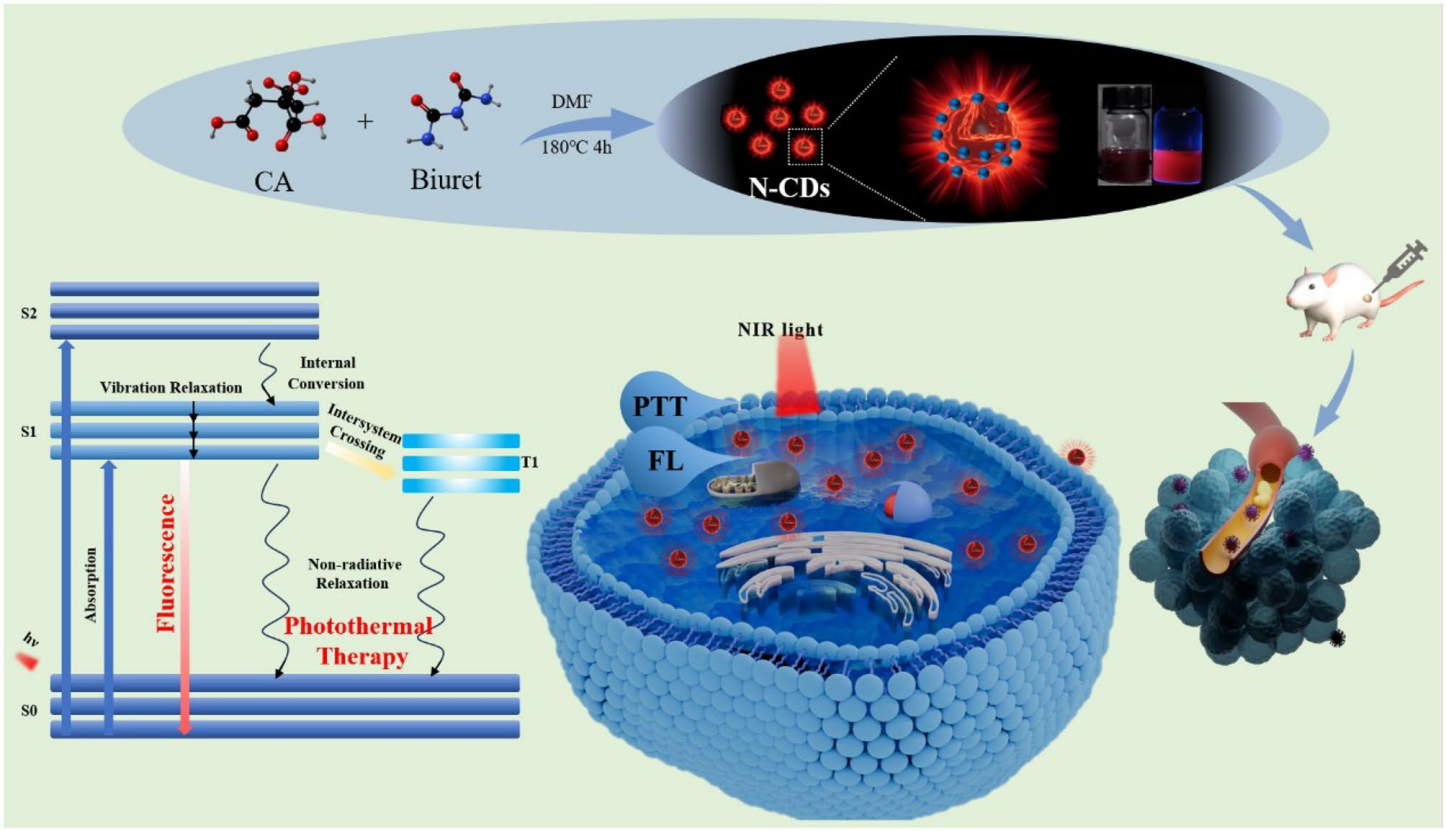
Schematically illustration of preparation and tumor ablation of N-CDs

In this study, as illustrated in Fig. 1, we developed nitrogen-doped carbon dots (N-CDs) through a facile one-pot hydrothermal synthesis using citric acid and biuret as precursors. The synthesized N-CDs exhibit exceptional photothermal stability, strong NIR-II absorption, and remarkable photothermal conversion efficiencies of 31.25% and 27.12% under 808 nm and 1060 nm laser irradiation, respectively. The fluorescence properties of the N-CDs are governed by their carbon core structure, surface states, and functional groups. By strategically expanding the sp² domains and enhancing surface oxidation and nitrogen doping, we successfully engineered the optical band of the N-CDs to achieve a red-shifted fluorescence emission [14, 31–33]. The resulting N-CDs demonstrate an optimal fluorescence emission peak at 660 nm, rendering them highly effective for bioluminescent imaging applications. Additionally, their superior photothermal properties make them promising candidates for antibacterial applications. With a compact particle size of approximately 4.8 nm and minimal toxicity, these N-CDs can efficiently penetrate biological systems and be safely excreted, underscoring their potential as biocompatible theranostic agents for integrated tumor diagnosis and therapy.

## Experimental section

### Synthesis of N-CDs

The nitrogen-doped carbon dots (N-CDs) were synthesized using an optimized method adapted from previously reported procedures [34]. Briefly, 2 g of citric acid and 4.5 g of biuret were dissolved in 30 mL of dimethylformamide (DMF) under continuous magnetic stirring to form a homogeneous solution. The mixture was then transferred into a 100 mL reaction flask and heated at 180 °C for 4 h. After cooling to room temperature, 40 mL of a NaOH solution (50 mg·mL^− 1^) was added to the reddish-brown suspension, followed by vigorous stirring until complete homogenization was achieved. The resulting mixture was centrifuged at 12,000 rpm for 10 min to isolate a black precipitate, which was subsequently washed three times with diluted hydrochloric acid and deionized water to remove impurities. Finally, the purified black powder was obtained through freeze-drying, yielding the N-CDs for further characterization and application.

### Photothermal effect

The photothermal performance of the material was systematically evaluated under irradiation with 808 nm and 1060 nm lasers at varying power densities (0.2 W·cm^− 2^, 0.4 W·cm^− 2^, 0.6 W·cm^− 2^, 0.8 W·cm^− 2^, and 1.0 W·cm^− 2^) and different concentrations (0.2 mg·mL^− 1^, 0.4 mg·mL^− 1^, 0.6 mg·mL^− 1^, 0.8 mg·mL^− 1^, and 1.0 mg·mL^− 1^). The temperature changes during irradiation were monitored and recorded in real-time using a high-resolution thermal imaging camera.

### Antibacterial activity

The antimicrobial effect of N-CDs against Staphylococcus aureus was evaluated using a plate-counting method. Bacterial suspensions were treated with N-CDs at specific concentrations and incubated under controlled conditions. Viable colonies were quantified by plating serial dilutions on agar and counting colony-forming units (CFUs). The antibacterial efficacy calculation, including the formula and experimental details, follows the methodology outlined in our prior publication [35].

### Cell experiment

#### In vitro cytotoxicity tests

N-CDs cytotoxicity was assessed via MTT assay. DU145 cells (1 × 10^4^ cells/well) were seeded in 96-well plates and incubated for 24 h (37 °C, 5% CO_2_). N-CDs were dissolved in medium at concentrations (0.2-1.0 mg·mL^− 1^), added to cells, and co-incubated for 4 h. Experimental groups were irradiated with 808–1060 nm lasers (1.0 W·cm^− 2^, 10 min), while controls were kept in the dark. After 24 h incubation, cells were stained with MTT for 4 h, followed by DMSO addition to dissolve formazan crystals. Absorbance at 492 nm was measured to quantify cell viability.

#### Cell uptake study

To investigate the cellular uptake of N-CDs, DU145 cells were seeded at 5 × 10^4^ cells/well in a 24-well plate and cultured for 24 h (37 °C, 5% CO_2_). N-CDs (1.0 mg·mL^− 1^) were sterilized and added to fresh medium, which replaced the existing medium. Cells were incubated for 4 h, washed with PBS, fixed with 4% paraformaldehyde for 15 min, and stained with DAPI for 10 min. Cellular uptake and distribution of N-CDs were analyzed using confocal microscopy.

#### Cell viability/dead cell study

To assess the anti-tumor efficacy of N-CDs, live-dead cell staining was conducted. DU145 cells were seeded in 24-well plates at 5 × 10^4^ cells/well and incubated for 24 h. The medium was replaced with fresh medium containing N-CDs (1.0 mg·mL^− 1^), and cells were co-incubated for 4 h to facilitate uptake. After washing with PBS, fresh medium was added. The experimental group was irradiated with 808 nm and 1060 nm lasers (1.0 W·cm^− 2^, 10 min), while controls were kept in the dark. Cells were then stained with Calcein-AM/PI for 30 min and imaged using confocal microscopy.

#### Apoptosis experiments

DU145 cells (5 × 10^4^ cells/well) were seeded in 24-well plates and cultured for 24 h. The medium was replaced with fresh medium containing N-CDs (1.0 mg·mL^− 1^) or PBS, and cells were incubated for 4 h. After washing with PBS, fresh medium was added. The experimental group was irradiated with 808–1060 nm lasers (1.0 W·cm^− 2^, 10 min), while the control group was kept in the dark. Cells were washed again, and 100 µL of 1×Binding Buffer was added to each well, followed by 5 µL of Annexin V-FITC and 10 µL of PI Staining Solution. After 15 min of dark incubation at room temperature, 400 µL of 1×Binding Buffer was added, and samples were imaged using confocal microscopy.

### Animal experiment

#### In vivo biosafety

To evaluate the long-term biosafety of N-CDs in vivo, a 14-day toxicity study was conducted in healthy mice. Healthy mice were intravenously injected with either PBS or N-CDs (5 mg·kg^− 1^). Serum and organ samples were collected on days 1, 7, and 14 post-injection for comprehensive biochemical analysis, inculding ALP, ALT, AST, CR, and UREA levels. Additionally, daily body weight measurements were recorded, and major organs were subjected to H&E staining and examined under a light microscope.

#### In vivo fluorescence imaging

Tumor-bearing nude mice were intratumorally injected with PBS or N-CDs (0.5 mg·kg^− 1^). Fluorescence imaging was performed at 1, 2, 4, 8, and 24 h post-injection using the IVIS Spectrometer Imaging System. After 24 h, the mice were euthanized, and fluorescence intensity in major organs and tumors was quantified.

#### In vitro antitumor study

Tumor-bearing nude mice were divided into six groups (three mice per group) to evaluate anti-tumor efficacy. Treatment included PBS, N-CDs alone, 808 nm laser only, 1060 nm laser only, N-CDs combined with 808 nm laser, and N-CDs combined with 1060 nm laser. Intratumoral injections of PBS or N-CDs (10 mg·kg^− 1^) were administered, followed by laser irradiation (1.0 W·cm^− 2^ 10 min). The temperature changes at the tumor site were monitored using a photothermal imaging system. Body weight and tumor volume (V = width^2^×length/2) were recorded every two days. After a 14-days, mice were euthanized, and tumors and major organs (heart, liver, spleen, lungs, kidneys) were excised for histopathological analysis via H&E staining.

#### Hemolysis reaction experiment

Blood from healthy mice was collected via orbital sinus puncture and stored in EDTA or citrate anticoagulant tubes. After gentle mixing and centrifugation (3000 rpm, 15 min), blood cells were isolated. N-CDs at various concentrations were mixed with blood cells, DI water, and PBS, incubated at 37 °C for 4 h, and centrifuged again. Hemolysis was visually assessed, and supernatant absorbance at 542 nm was measured using a microplate reader.

$$\eqalign{& {\rm{Hemolysis}}\,{\rm{Rate }}\left( \% \right) \cr &:{{OD ({\rm{sample)}} - OD ({\rm{PBS)}}} \over {OD ({\rm{ddH2O)}} - OD ({\rm{PBS)}}}}\,\, \times 100\% \cr} $$

#### Statistical analysis

All experiments were conducted in triplicate, with results expressed as mean ± standard deviation.

## Results and discussion

### Characterization of N-CDs

**Fig. 2:**
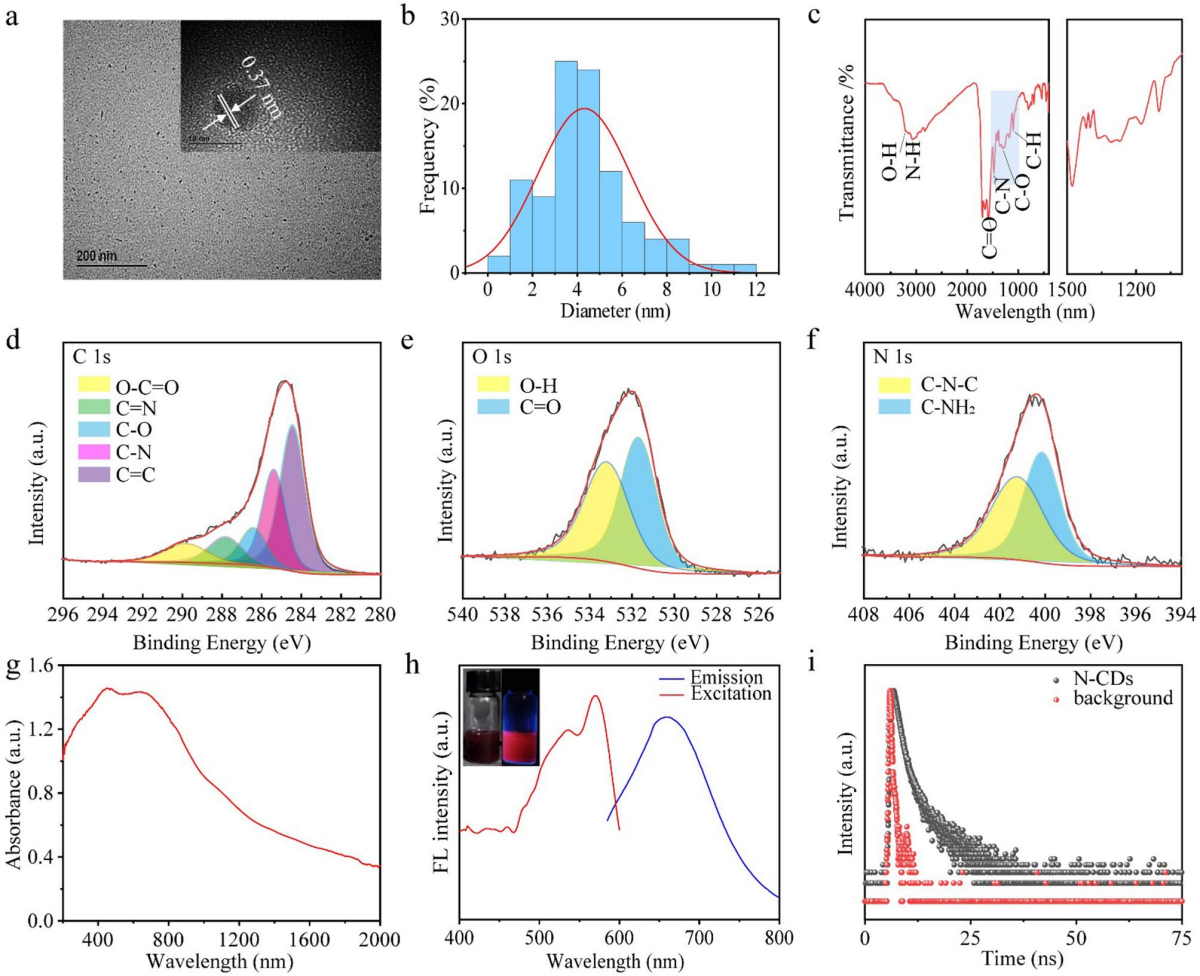
(**a**) Transmission Electron Microscopy (TEM) image of N-CDs. (**b**) Particle size distribution curve for N-CDs. (**c**) Fourier Transform Infrared (FTIR) spectrum of N-CDs. (**d**-**f**) XPS spectra of N-CDs, C element (**d**), O element (**e**), N element (**f**). (**g**) UV-vis-NIR spectrum of N-CDs. (**h**) Fluorescence emission spectrum of N-CDs. (**i**) Fluorescence decay curve of N-CDs in aqueous solution

The transmission electron microscopy (TEM) image of N-CDs (Fig. 2a) reveals spherical zero-dimensional structures, with the high-resolution TEM image (Fig. 2a inset) demonstrating a lattice structure with an interplanar spacing of about 0.37 nm. This spacing aligns with d-spacing of the graphitic carbon (002) lattice plane [36]. Analysis in Fig. 2b indicates that the average particle size of N-CDs is about 4.8 nm. The X-ray diffraction (XRD) pattern (Fig. S1) exhibits a distinct peak at roughly 27°, corresponding to the typical (002) diffraction of graphitic carbon, indicative of a crystalline, layered structure similar to graphene. The XRD data confirm a well-defined graphitic structure, consistent with previous reports on N-doped CDs, where graphitic nitrogen doping induces shifts in absorption and emission spectra [37]. Specifically, graphitic nitrogen narrows the energy gap between the highest occupied molecular orbital (HOMO) and the lowest unoccupied molecular orbital (LUMO), leading to a red shift in the spectral response [12].

In the Fourier transform infrared (FT-IR) spectra (Fig. 2c), the absorption peaks at 3209 and 3080 cm^− 1^ for N-CDs correspond to O-H and N-H groups, respectively [37]. The peak at 1580 cm^− 1^ is attributed to C = O absorption, while the peak at 1477 cm^− 1^ arises from the asymmetric stretching vibration of C-N. Additionally, the absorption peak at 1309 cm^− 1^ is associated with the stretching vibration of C-O [36].

The surface chemical properties of CDs were further investigated using X-ray Photoelectron Spectroscopy (XPS). The full spectrum (Fig. S2) revealed three major peaks at 289.7, 399.2, and 531.7 eV, corresponding to the elements C, N, and O, respectively. The elemental composition of N-CDs was calculated to be 74.66% of C, 11.23% of N and 14.11% of O, respectively (Table S1). The high-resolution C1s spectrum (Fig. 2d) was deconvoluted into five peaks at 284.5, 285.6, 286.6, 287.9, and 289.8 eV, which correspond to C = C, C-N, C-O, C = N, and O-C-O functional groups, respectively [38, 39]. The O1s spectrum (Fig. 2e) displayed two peaks at 531.5 and 533.4 eV, attributed to O-H and C = O groups, respectively [40]. The N1s spectrum (Fig. 2f) revealed peaks at 400.2 eV and 401.5 eV, corresponding to C-N-C and C-NH_2_, bonds, respectively, indicative of pyrrole nitrogen and graphitic nitrogen [9]. The XPS findings are consistent with FT-IR spectroscopy results, under high-pressure and high-temperature conditions, the carboxyl groups of citric acid react with the amine groups of biuret through amide condensation, effectively incorporating nitrogen into CDs. In the doped system, the introduction of graphitic nitrogen induces a red shift in visible light absorption into the near-infrared spectral range. This red shift is attributed to the presence of graphitic nitrogen, which narrows the H-L gap, with the extent of the shift increasing with higher nitrogen doping concentrations [33]. Graphitic nitrogen doping creates mid-gap states within the original H-L gap of an undoped system, a result of excess electrons being donated into the unoccupied π* orbitals of the conjugated system [41]. The fluorescence properties of the graphitic nitrogen doped system exhibit a significant narrowing of the H-L gap, leading to a pronounced red-shifted emission compared to the undoped system [33]. The Raman spectra of N-CDs (Fig. S3) showed two broad peaks corresponding to the D band and G band at about 1350 cm^− 1^ and 1615 cm^− 1^, respectively. The relative intensities of the D band to the G band (ID/IG = 0.95) indicated a high carbon lattice structures in the N-CDs content.

The optical properties of the N-CDs were charaterized to evaluate their fluorescence performance. As shown in the UV-vis-NIR absorption spectrum (Fig. 2g), the N-CDs exhibited a broad absorption range from 400 nm to 2000 nm, with strong absorption in the NIR-II region, indicating their exceptional NIR light absorption capabilities. This superior NIR absorption is attributed to the high content of graphitic nitrogen doping [33]. UV-vis-NIR spectrum of the N-CDs in solution (Fig. S4) further corroborates their strong absorption in the NIR-II region. As shown in Fig. 2h, Under excitation at 565 nm, the N-CDs exhibited fluorescence emission with a peak at 660 nm (Fig. 2h), which lies within the NIR-I biological window, enabling deeper fluorescence penetration. The fluorescence decay curve of N-CDs (Fig. 2i) revealed a fluorescence lifetime of 37.72 ns. Furthermore, the emission intensity of N-CDs varied with the excitation wavelength (Fig. S5), demonstrating excitation-dependent emission behavior, likely due to the presence of multiple emission centers on the surface of the N-CDs [27].

### Photothermal performance

**Fig. 3:**
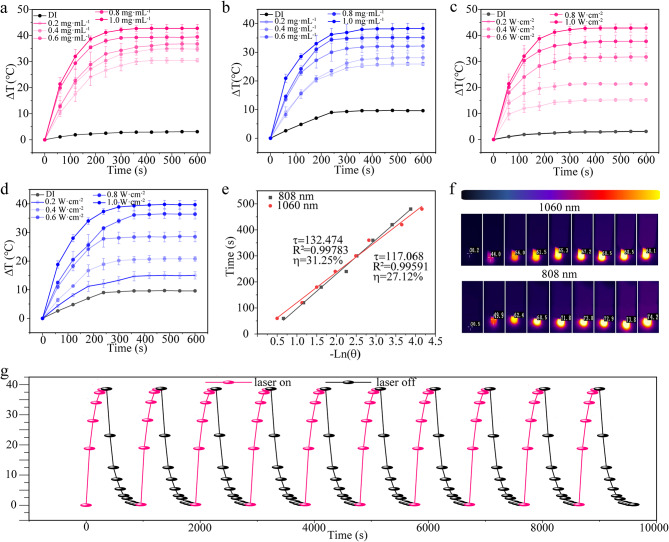
(**a**-**d**) Photothermal heating curves of N-CDs, showing the effect of different concentrations (**a**-**b**), different powers (**c**-**d**). (**e**) Linear relationship between -lnθ and time. (**f**) Effect of heating of N-CDs under the thermal imaging camera. (**g**) Ten photothermal cycles of N-CDs (1.0 mg·mL^− 1^) under 1060 nm laser illumination (1.0 W·cm^− 2^)

The UV-vis-NIR spectrum of the N-CDs revealed broad absorption peaks in both NIR-I and NIR-II regions, highlighting their potential as effective near-infrared photothermal agents. To evaluate their photothermal performance, laser sources at 808 nm and 1060 nm were employed. The study initially investigated the correlation between temperature rise and concentration of N-CDs under 808 nm and 1060 nm laser irradiation (1.0 W·cm^− 2^). As shown in Fig. 3a, DI water displayed a minimal temperature increase of only 5 ℃ under 808 nm irradiation. In contrast, a 0.2 mg·mL^− 1^ solution of N-CDs showed a temperature rise of 29 ℃, while solutions at 0.4 mg·mL^− 1^ and 0.6 mg·mL^− 1^ resulted in increases of 34 ℃ and 38 ℃, respectively. The highest concentrations, 0.8 mg·mL^− 1^ and 1.0 mg·mL^− 1^, exhibited temperature rises of 40 ℃ and 45 ℃, respectively. A similar trend was observed under 1060 nm laser irradiation (Fig. 3b), where DI water increased by only 9 ℃, while the 0.2 mg·mL^− 1^ N-CDs solution rose by 24 ℃. Solutions at concentrations of 0.4, 0.6, 0.8, and 1.0 mg·mL^− 1^ demonstrated temperature rises of 28 ℃, 32 ℃, 34 ℃, and 38 ℃, respectively. These results clearly demonstrate the concentration-dependent photothermal effect of N-CDs.

The relationship between the temperature rise of N-CDs (1.0 mg·mL^− 1^) and laser power at 808 nm and 1060 nm was also examined. As shown in Fig. 3c, under 808 nm laser irradiation, the temperature increases of 18 ℃, 21 ℃, 34 ℃, 38 ℃, and 43 ℃ were observed at power densities of 0.2, 0.4, 0.6, 0.8, and 1.0 W·cm^− 2^, respectively. Similarly, under 1060 nm laser irradiation (Fig. 3d), the temperature increases of 13 ℃, 19 ℃, 26 ℃, 33 ℃, and 39 ℃ were recorded at the same power densities. Additionally, based on the cooling phase data, the photothermal conversion efficiency of N-CDs was calculated to be 31.25% under 808 nm irradiation and 27.12% under 1060 nm irradiation (Fig. 3e). These values suggest that the N-CDs exhibit high photothermal conversion efficiency, making them highly effective as photothermal agents for therapeutic applications in tumor treatment under NIR light.

Moreover, Fig. 3f shows the infrared thermal imaging of the N-CDs solution (1.0 mg·mL^− 1^) under both 808 nm (1.0 W·cm^− 2^) and 1060 nm (1.0 W·cm^− 2^) laser illumination. Significant temperature increases were observed at each time interval (0, 2, 4, 6, 8, and 10 min), highlighting the exceptional photothermal efficiency of N-CDs. This efficiency can be attributed to several factors. Firstly, the amide condensation reaction between carboxyl and amine groups results in a nitrogen-rich surface, particularly the incorporation of graphitic nitrogen, which shifts the optical bandgap of N-CDs toward longer wavelengths [29]. Additionally, smaller nanoparticles exhibit higher light-to-heat conversion efficiency due to their increased surface area and reduced light scattering, minimizing energy loss during irradiation. Finally, the excellent solubility and dispersibility of N-CDs in water facilitate rapid heat transfer to the surrounding medium, further enhancing temperature rise [9].

Additionally, the photothermal stability of N-CDs was assessed through ten heating and cooling cycles. As shown in Fig. 3g and S6, the heating-cooling cycle tests demonstrated that the N-CDs maintained consistent photothermal performance across ten cycles under both 1060 nm and 808 nm illumination. No significant decline in photothermal performance was observed, confirming the excellent photothermal stability of N-CDs under NIR light exposure.

Moreover, the suspension stability of nanoparticles is determined by their Zeta potential. The Zeta potentials of N-CDs were measured to be -16.01 mV in DI, -30.53 mV in PBS, and − 26.79 mV in RPMI-1640 medium (Fig. S7). Nanoparticles with a higher surface charge, as indicated by Zeta potential, exhibit stronger repulsive force, leading to stable dispersion and retention [42].

### Antibacterial of N-CDs

Given their strong photothermal effects in both the NIR-I and NIR-II regions, the near-infrared antibacterial capabilities of N-CDs were evaluated using *Staphylococcus aureus* as a model organism. The antibacterial performance was tested at various N-CDs concentrations (0.2, 0.4, 0.6, 0.8, and 1.0 mg·mL^− 1^) and different laser power densities (0.2, 0.4 0.6, 0.8, and 1.0 W·cm^− 2^). As shown in Fig. S8 a and b, at fixed laser power of 1.0 W·cm^− 2^ illumination, the number of bacterial colonies dramatically decreased as the concentration of the N-CDs solution increased, reaching maximum antibacterial efficiency of 95% and 90% at 808 and 1060 nm, respectively. Furthermore, the relationship between antibacterial efficacy and laser power was also studied. As depicted in Fig. S8 c and d, as the power increased to 1.0 W·cm^− 2^, the antibacterial efficiency reached 94% and 90% under 808 and 1060 nm irradiation, respectively. These results confirm that N-CDs effectively absorb NIR light and convert it into thermal energy, generating sufficient heat to inhibit or eliminate *Staphylococcus aureus*.

### Cellular experiments

**Fig. 4:**
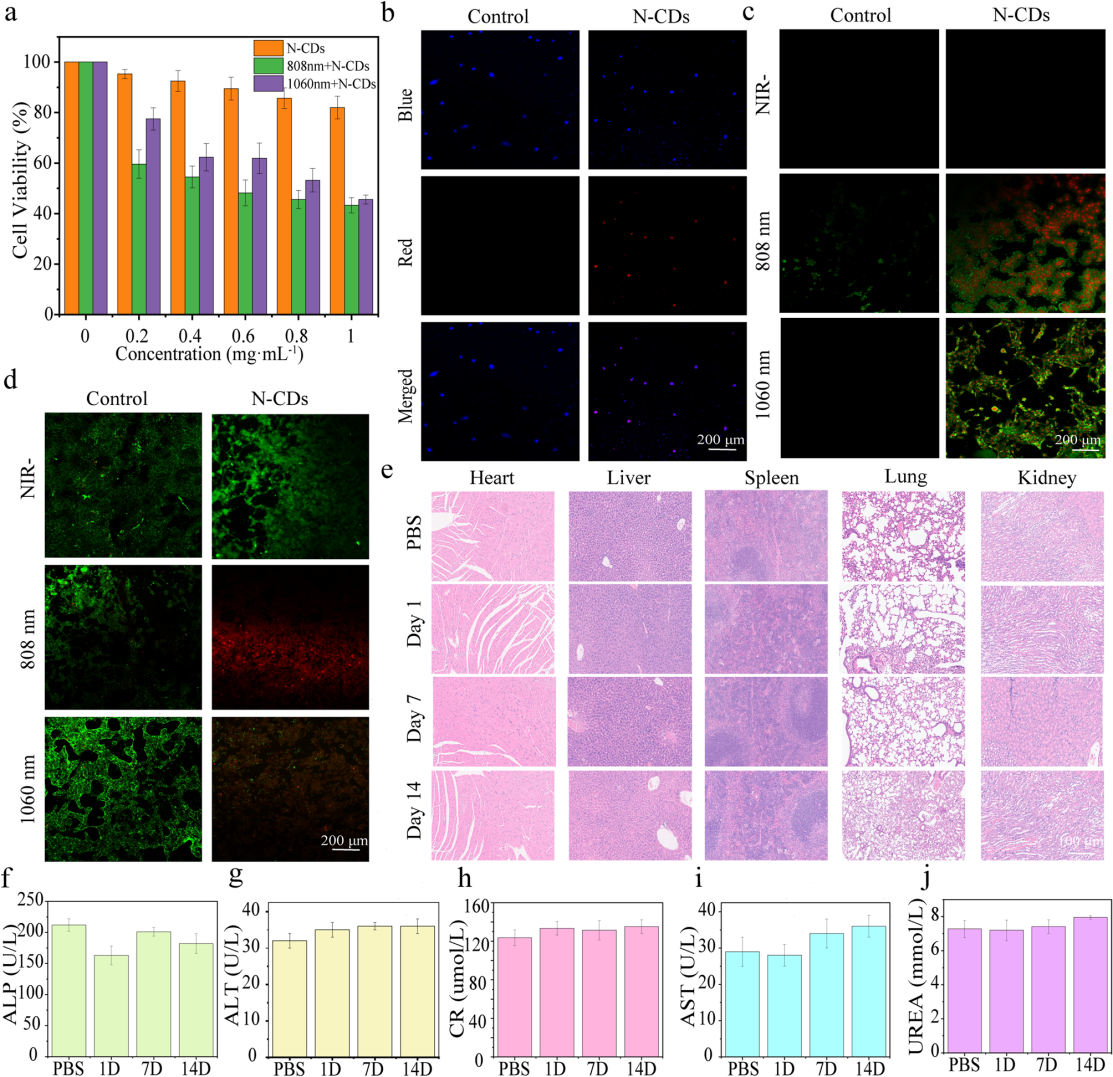
(**a**) Viability of DU145 cells incubated with solutions containing different concentrations of N-CDs under 808 nm and 1060 nm laser irradiation. (**b**) Uptake of N-CDs by DU145 cells incubated in PBS (control group) and N-CDs solution (experimental group). (**c**) Apoptosis imaging of DU145 cells incubated in PBS (control group) and N-CDs solution (experimental group) under dark conditions, with 1060 nm and 808 nm laser irradiation. (**d**) Live/dead imaging of DU145 cells incubated in PBS (control group) and N-CDs solution (experimental group) under dark conditions, with 1060 nm and 808 nm laser irradiation. (**e**) H&E staining of major organs (heart, lung, kidney, liver, and spleen) from healthy mice 1, 7, and 14 days after intravenous injection of PBS and N-CDs. (**f**)-(**j**) Values of major functional factors (alanine aminotransferase, ALT; alkaline phosphatase, ALP; aspartate aminotransferase, AST) in the liver, serum creatinine (CR), and urea (UREA) in healthy mice 1, 7, and 14 days after intravenous injection of PBS and N-CDs

The PTT performance of N-CDs at the cellular level was evaluated using DU145 cells. Initially, the cytotoxicity of N-CDs was assessed using the MTT assay [9]. To avoid thermal damage to healthy tissues, the laser power density was maintained at 1.0 W·cm^− 2^ throughout the experiment. Notably, cell viability remained high even at a N-CDs concentration of 1.0 mg·mL^− 1^ without laser irradiation, indicating low intrinsic cytotoxicity (Fig. 4a). However, when subjected to 808–1060 nm lasers irradiation, cell viability decreased significantly. This suggests that N-CDs exhibit considerable phototoxicity under NIR light, attributed to their excellent photothermal conversion ability, underscoring their potential for effective photothermal therapy.

In addition, the cellular uptake ability of the N-CDs was evaluated using laser confocal scanning microscopy. As depicted in Fig. 4b, the extensive overlap between the blue fluorescence (DAPI-stained nuclei) and the red fluorescence (internalized N-CDs) strongly suggests that the N-CDs were efficiently internalized by the cells rather than merely adhering to their surface. This efficient internalization further highlights the significant potential of N-CDs in tumor-targeted therapies.

Annexin V-FITC and Propidium Iodide (PI) staining were utilized to assess cell apoptosis (Fig. 4c). Early apoptotic cells were identified by green fluorescence, while late apoptotic cells exhibited both green and red fluorescence. In the control group, minimal apoptosis was observed under all conditions. In contrast, the experimental group treated with N-CDs showed a marked increase in apoptosis upon laser exposure, with most cells displaying dual green and red fluorescence under both 808 nm and 1060 nm illumination. These results confirm the significant phototoxic effects of N-CDs on cells under NIR light exposure, highlighting their considerable potential for photothermal therapy applications.

The in vitro photothermal therapeutic efficacy of the N-CDs was further validated using a dual-staining experiment using Acridine Orange (AO) and Propidium Iodide (PI) dyes. In this assay, live cells were stained green by AO, while dead cells appeared red due to PI staining. As illustrated in Fig. 4d, the control group, treated with PBS, exhibited predominantly green fluorescence, indicating minimal cellular damage. In contrast, cells treated with N-CDs and exposed to the same conditions demonstrated pronounced red fluorescence at both 808 nm and 1060 nm wavelengths, indicting substantial cell death. These findings align with the results from the MTT assay, further confirming effective photothermal therapeutic potential of the N-CDs.

To further assess the long-term in vivo biocompatibility of N-CDs, healthy mice were administered a dose of 5.0 mg·kg^− 1^. Histological analysis of major organs (heart, lungs, kidneys, liver, and spleen) was conducted using H&E staining. The results indicted no significant adverse effects in mice treated with N-CDs solution over the first 14 days (Fig. 4e). Additionally, liver functional markers, including alanine aminotransferase (ALT), alkaline phosphatase (ALP), aspartate aminotransferase (AST), serum creatinine (CR), and urea (UREA) level, showed no notable differences between the control and experimental groups (Fig. 4f-j).

Furthermore, no significant changes in the body weight of the mice were observed during the treatment period (Fig. S9). The biocompatibility of N-CDs was further supported by coagulation tests (Fig. S10). Mouse blood exposed to PBS or the N-CDs solution maintained normal coagulation capability, whereas exposure to water resulted in hemolysis and loss of coagulation ability, highlighting the safe interaction between N-CDs and biological systems. Taken together, these findings provide compelling evidence that N-CDs exhibit high biocompatibility, making them suitable for PTT.

### Animal experiments

**Fig. 5:**
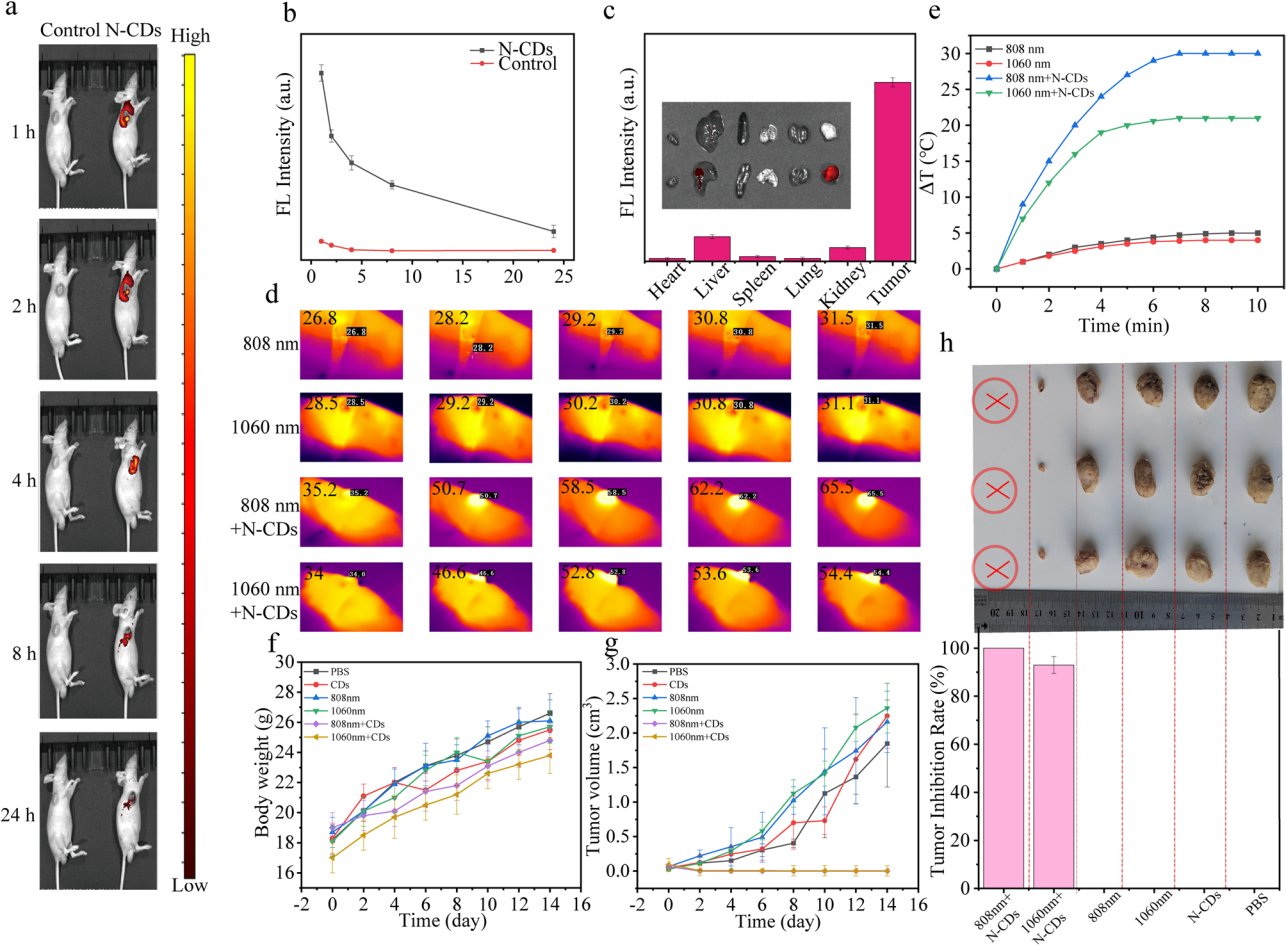
(**a**) Fluorescence visualization of DU145 tumor-bearing nude mice subsequent to the administration of the injection of N-CDs at corresponding time points, with the red circle indicating the tumor area. (**b**) Fluorescence photographs of the major organs and tumor in DU145 tumor-bearing nude mice taken 24 h post-injection with N-CDs, along with the fluorescence intensities. (**c**) Fluorescence intensity values at the tumor site of Nude mice with DU145 tumors at various time intervals following the injection of N-CDs. (**d**) Infrared thermograms of the tumor sites in DU145 tumor-bearing nude mice from the control and experimental groups under 808–1060 nm (1.0 W·cm^− 2^) irradiation at different time intervals, and (**e**) the resultant temperature increase. (**f**) Weight change curves of tumor-bearing nude mice over 14 days under different treatments. (**g**) Tumor volume change curves of tumor-bearing nude mice over 14 days under different treatments. (**h**) Schematic illustrations of tumors in different groups of tumor-bearing nude mice after 14 days

To assess the potential of N-CDs as a theranostic agent, their in vivo fluorescence imaging capabilities were further examined. Nude mice bearing subcutaneous DU145 tumors were selected as the animal model, and N-CDs were administered via intratumoral injection. As depicted in Fig. 5a and b, the fluorescence intensity at the tumor site progressively increased over time, peaking approximately 1 h post-injection before gradually declining. This indicates that the uptake of N-CDs by tumor cells reaches its maximum within 1 h. By 24 h, the fluorescence signal had nearly disappeared, suggesting that the N-CDs were extensively metabolized and cleared from the tumor site. Following intratumoral injection of N-CDs solution in mice, we performed quantitative analysis of fluorescence intensity in the tumor tissue. As shown in Table. S2, the fluorescence intensity of N-CDs reached 9.092 × 10^7^ at 1 h post-injection, which subsequently decreased to 1.39 × 10^7^ after 24 h, yielding a ratio of approximately 0.153. This indicates an 84.7% reduction of N-CDs at the tumor site within 24 h, demonstrating substantial metabolic clearance. The dose of N-CDs was calculated to be injected into the mouse, resulting in a total mass of 10 µg. After 24 h, the metabolic rate was 84.7%, thus, only 1.53 µg of N-CDs remained within the tumor of the mouse.

Additionally, to more precisely assess the biodistribution of N-CDs, the mice were humanely euthanized 24 h post-injection, and their major organs (heart, liver, spleen, lung, and kidney) as well as the tumor were collected for further analysis. The fluorescence images and quantified fluorescence intensity values of the excised tissues (Fig. 5c) revealed minimal signals in major organs, indicating efficient metabolism of N-CDs. Importantly, the fluorescence in the tissue samples was primarily within the 500–600 nm range. Notably, the red fluorescence emitted by N-CDs at 660 nm provides a significant advantage. This specific red emission effectively reduces interference from intrinsic tissue autofluorescence, positioning N-CDs as a highly promising candidate for live imaging applications.

Given the excellent photothermal properties and high biocompatibility of N-CDs, their in vivo antitumor performance was assessed under NIR irradiation (Fig. S11). Tumor-bearing mice were randomly divided into six groups and treated with PBS, N-CDs alone, 808 nm laser alone, 1060 nm laser alone, N-CDs + 808 nm laser, and N-CDs + 1060 nm laser, respectively. To minimize potential photodamage to healthy tissue, the laser power density was maintained at a relatively low of 1.0 W·cm^− 2^. The infrared thermograms and temperature changes at the tumor sites were monitored using an infrared camera. As shown in Fig. 5d and e, the N-CDs + 808 nm and N-CDs + 1060 nm groups exhibited a significant temperature increase at the tumor sites, while the groups receiving only laser irradiation (808–1060 nm) showed minimal temperature changes. After 4 min of irradiation, the tumor temperature in the N-CDs + 808 nm and N-CDs + 1060 nm groups rose from 35.2 °C to 58.5 °C and from 34.0 °C to 52.8 °C, respectively. In contrast, the 808 nm and 1060 nm control groups showed negligible temperature changes (approximately 4.7 °C and 2.6 °C, respectively). In addition, no significant fluctuations in body weight were observed during the 14-day treatment period (Fig. 5f), indicating that N-CDs cased minimal systemic side effects.

As shown in Fig. 5g and h, the tumors in N-CDs + 808 nm group were completely eradicated, while those in the N-CDs + 1060 nm group were nearly ablated. In contrast, tumors in control groups continued to grow rapidly, visually confirming the superior tumor growth inhibition in the N-CDs + 808 nm and N-CDs + 1060 nm treatments. Furthermore, no tumor recurrence was observed during the follow-up period, demonstrating the effectiveness of N-CDs in preventing tumor regrowth.

Subsequently, histological evolution of major organs (heart, lungs, kidneys, liver, and spleen) from the treated mice revealed no significant pathological changes in either the control or experimental groups. This finding further supports the safety profile of N-CDs for vivo applications (Fig. S12).

Furthermore, histological examination of tumor tissue sections from the treatment groups revealed pronounced signs of apoptosis and necrosis, whereas the control group exhibited no significant cellular damage (Fig. S13). These observations underscore the robust photothermal therapeutic efficacy of N-CDs, highlighting their potential as a promising and safe modality for cancer treatment.

## Conclusions

In summary, we have successfully developed nitrogen-doped carbon dots (N-CDs) with strong absorption across both NIR-I and NIR-II regions, coupled with superior red fluorescence emission. These properties make N-CDs highly efficiency as photothermal agents for fluorescence-guided imaging in the NIR biological windows. The N-CDs feature uniform size distribution (4.8 nm), excellent water solubility, dispersibility, biocompatibility, and photostability, with impressive photothermal conversion efficiencies of 31.25% (808 nm) and 27.12% (1060 nm). The N-CDs also display remarkable antibacterial activity in vitro and robust fluorescence imaging capabilities in vivo. The N-CDs exhibit efficient cellular uptake and are effectively excreted through the filtration systems of body, minimizing concerns about long-term biotoxicity. In vitro and in vivo studies confirm potent antitumor effects under low-power laser irradiation, highlighting their potential as a versatile cancer therapeutic platform. This scalable synthesis approach paves the way for developing multifunctional photothermal agents for effective tumor therapy.

## Electronic supplementary material

Below is the link to the electronic supplementary material.

Supplementary material 1

Supplementary Material 2

## Data Availability

No datasets were generated or analysed during the current study.
