# Correction: N-doped carbon dots for dual-modality NIR fluorescence imaging and photothermal therapy

**DOI:** 10.1186/s12951-025-03662-x

**Published:** 2025-08-29

**Authors:** Hui-Xian Shi, Xuan Qu, Tong-Tong Zhao, Zhong-Fu An, Chuan-Yi Zhang, Hong-Liang Wang

**Affiliations:** 1 Shanxi Key Laboratory of Biomedical Metal Materials, College of Materials Science and Engineering, Taiyuan University of Technology, Taiyuan, 030024 China; 2 Key Laboratory of Flexible Electronics (KLOFE) & Institute of Advanced Materials (IAM), Nanjing Tech University, 30 South Puzhu Road, Nanjing, 211816 China; 3 Department of Nuclear Medicine, First Hospital of Shanxi Medical University, Taiyuan, 030001 Shanxi China; 4 Shanxi Key Laboratory of Molecular Imaging & Collaborative Innovation Center for Molecular Imaging of Precision Medicine, Shanxi Medical University, Taiyuan, 030001 Shanxi China

**Correction: Journal of Nanobiotechnology (2025) 23:513** 10.1186/s12951-025-03497-6

In this article Figs. 3, 4 and 5 appeared incorrectly and have now been corrected in the original publication. For completeness and transparency, the old incorrect and corrected versions of these figures are displayed below.

For these figures, the author conducted multiple additional repeat experiments and added error bars to the data. The images themselves were also reacquired using a higher-resolution confocal microscope. All these changes were made to ensure the data presented is more precise and the article is of the highest quality possible.

Incorrect Fig. 3.


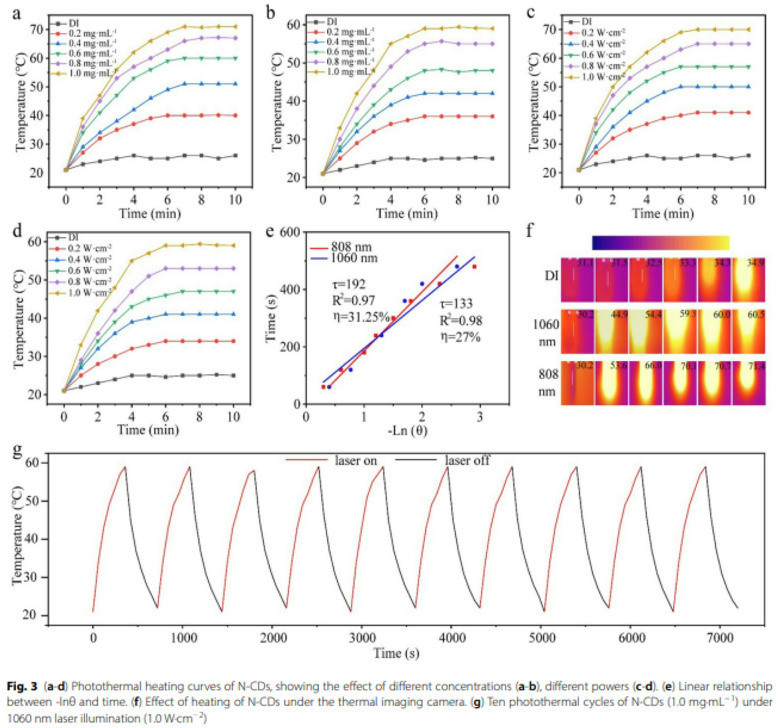


Correct Fig. 3.

**Fig. 3:**
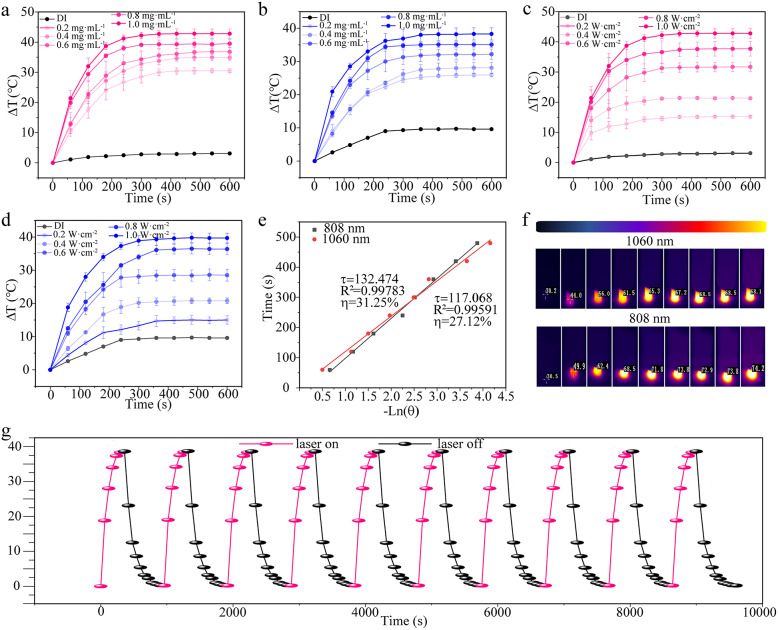
(**a**-**d**) Photothermal heating curves of N-CDs, showing the effect of different concentrations (**a**-**b**), different powers (**c**-**d**). **e** Linear relationship between -lnθ and time. **f** Effect of heating of N-CDs under the thermal imaging camera. **g** Ten photothermal cycles of N-CDs (1.0 mg·mL^−1^) under 1060 nm laser illumination (1.0 W·cm^−2^)

Incorrect Fig. 4.


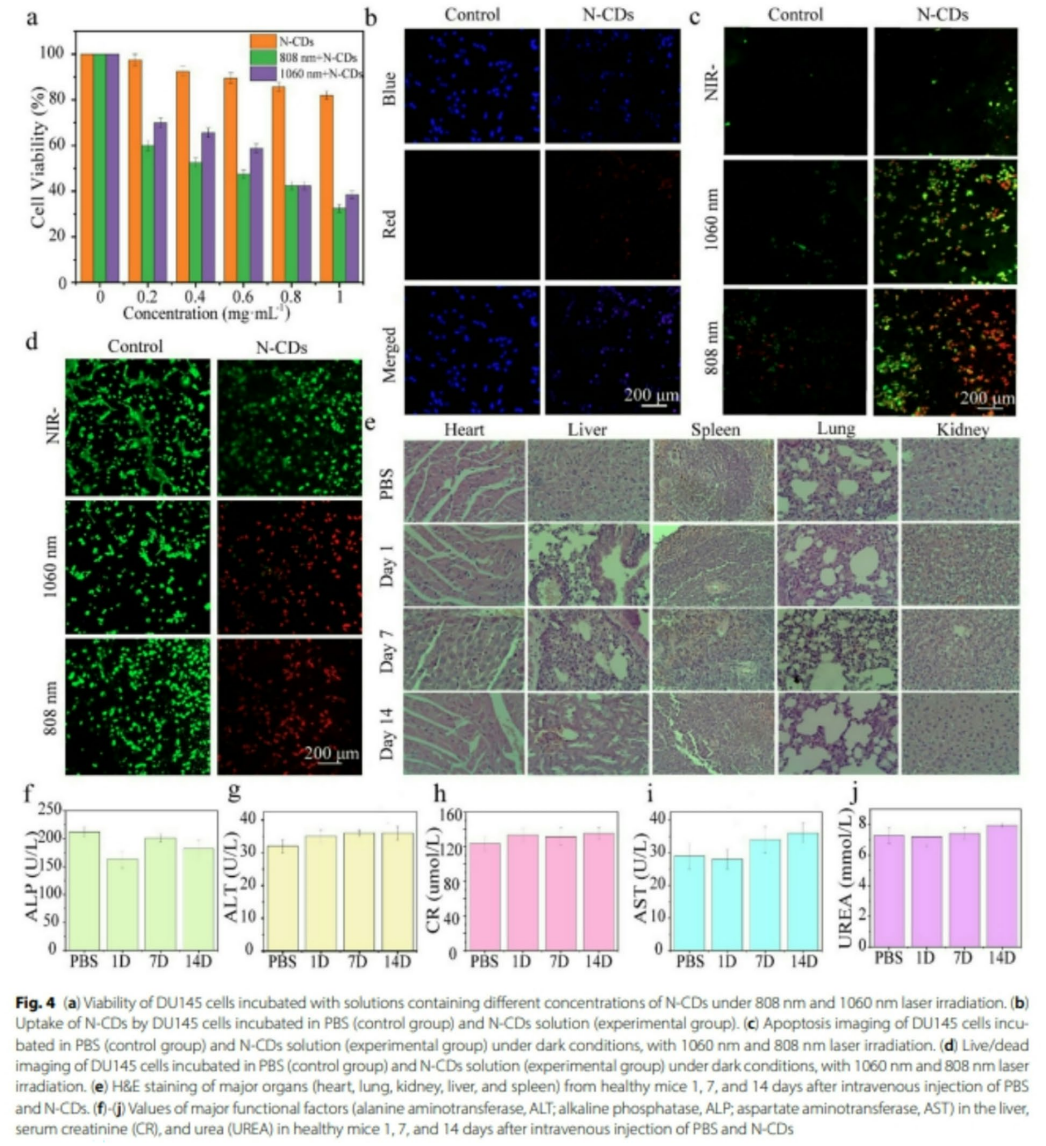


Correct Fig. 4.

**Fig. 4:**
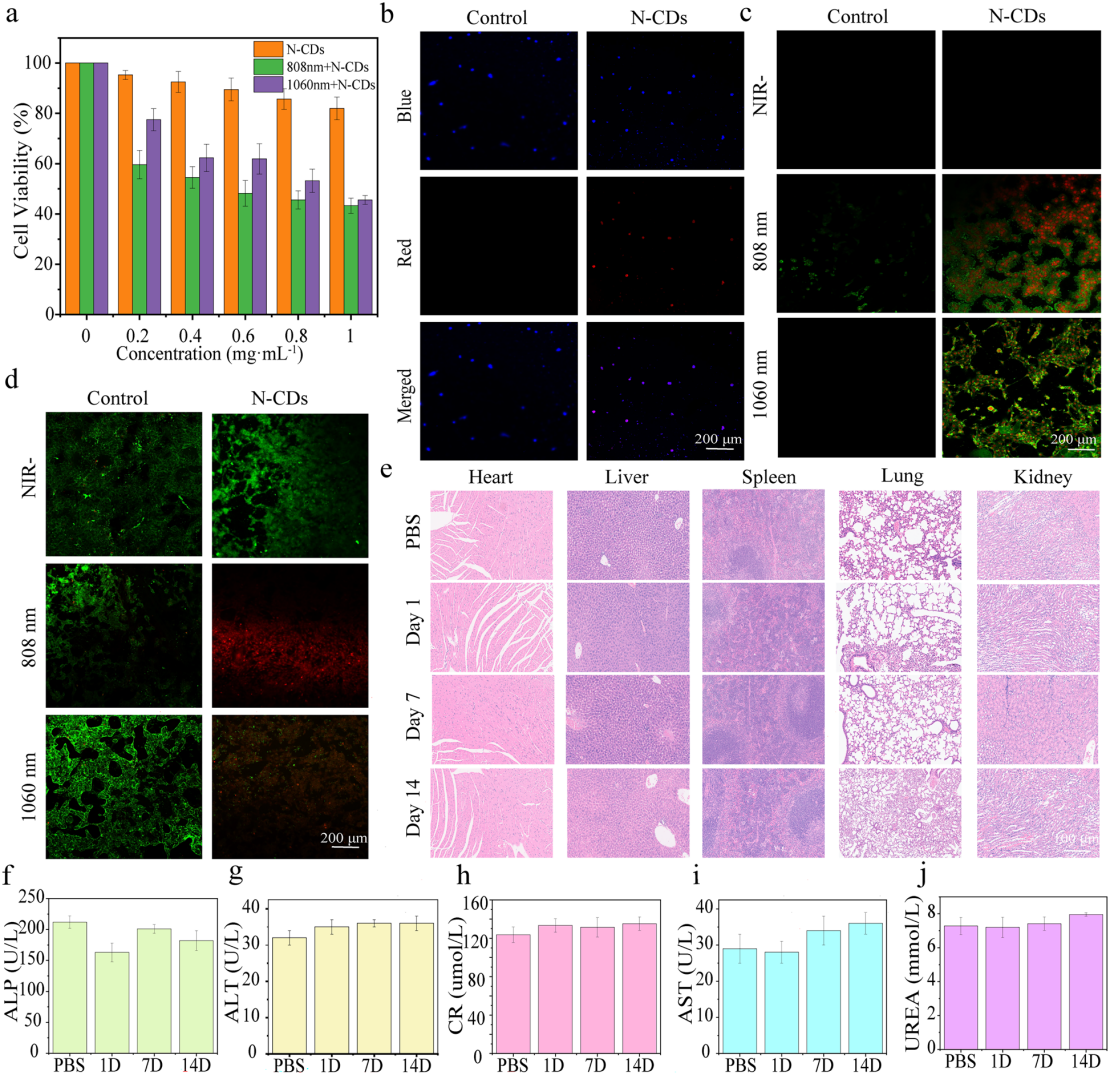
(**a**) Viability of DU145 cells incubated with solutions containing different concentrations of N-CDs under 808 nm and 1060 nm laser irradiation. **b** Uptake of N-CDs by DU145 cells incubated in PBS (control group) and N-CDs solution (experimental group). **c** Apoptosis imaging of DU145 cells incubated in PBS (control group) and N-CDs solution (experimental group) under dark conditions, with 1060 nm and 808 nm laser irradiation. **d** Live/dead imaging of DU145 cells incubated in PBS (control group) and N-CDs solution (experimental group) under dark conditions, with 1060 nm and 808 nm laser irradiation. **e** H&E staining of major organs (heart, lung, kidney, liver, and spleen) from healthy mice 1, 7, and 14 days after intravenous injection of PBS and N-CDs. **f**-**j** Values of major functional factors (alanine aminotransferase, ALT; alkaline phosphatase, ALP; aspartate aminotransferase, AST) in the liver, serum creatinine (CR), and urea (UREA) in healthy mice 1, 7, and 14 days after intravenous injection of PBS and N-CDs

Incorrect Fig. 5.


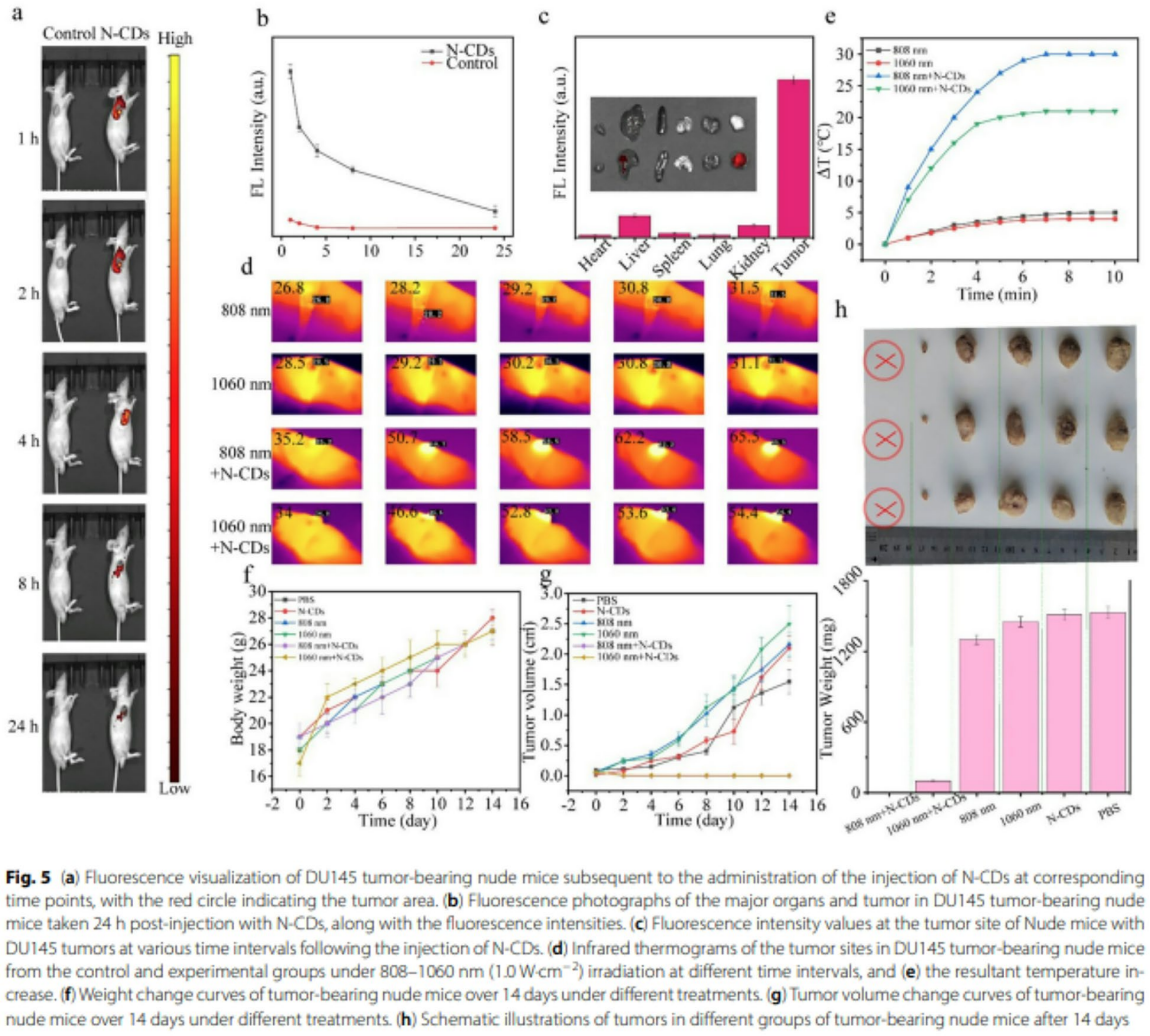


Correct Fig. 5.

**Fig. 5:**
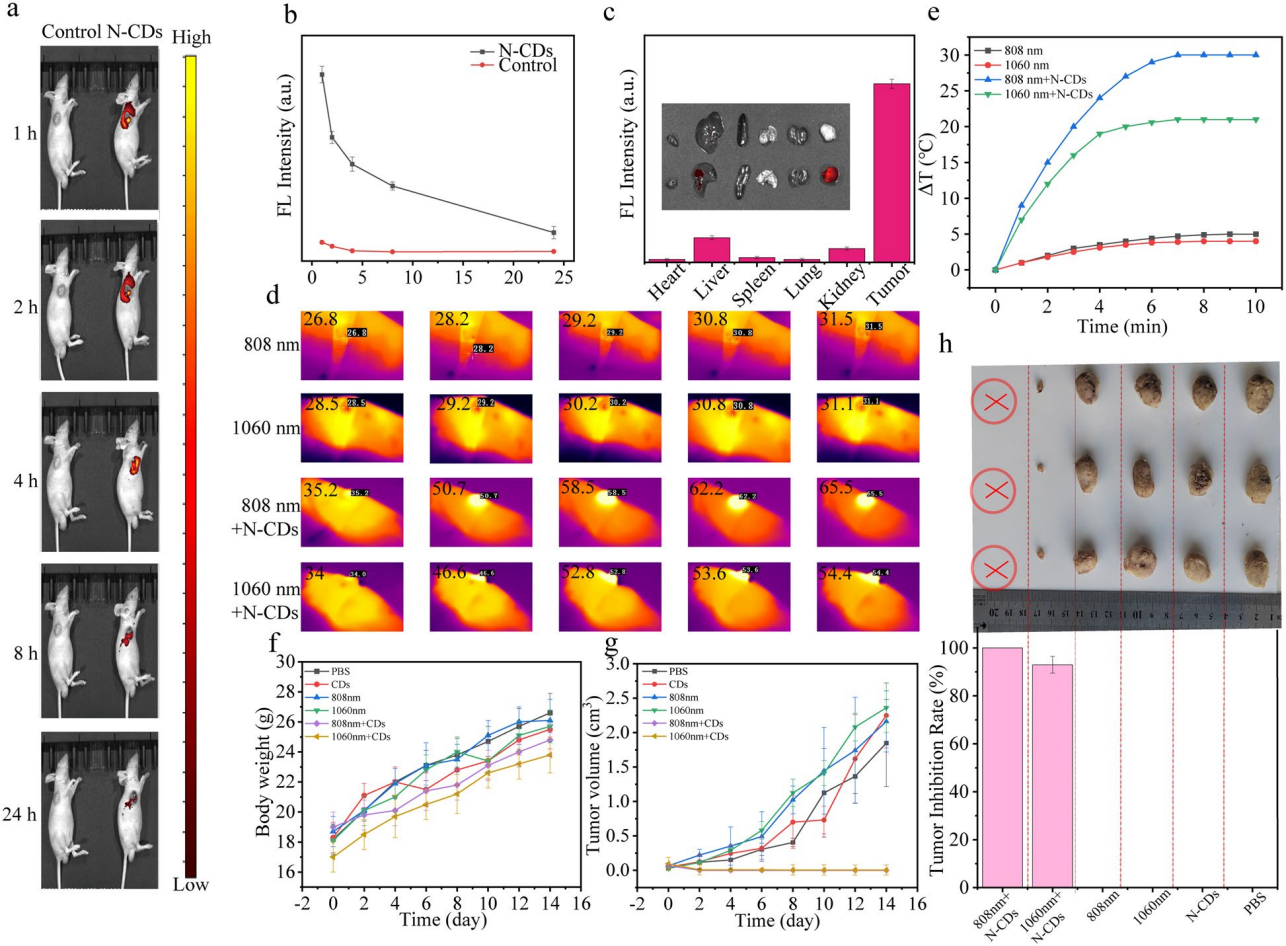
(**a**) Fluorescence visualization of DU145 tumor-bearing nude mice subsequent to the administration of the injection of N-CDs at corresponding time points, with the red circle indicating the tumor area. **b** Fluorescence photographs of the major organs and tumor in DU145 tumor-bearing nude mice taken 24 h post-injection with N-CDs, along with the fluorescence intensities. **c** Fluorescence intensity values at the tumor site of Nude mice with DU145 tumors at various time intervals following the injection of N-CDs. **d** Infrared thermograms of the tumor sites in DU145 tumor-bearing nude mice from the control and experimental groups under 808–1060 nm (1.0 W·cm^−2^) irradiation at different time intervals, and (**e**) the resultant temperature increase. **f** Weight change curves of tumor-bearing nude mice over 14 days under different treatments. **g** Tumor volume change curves of tumor-bearing nude mice over 14 days under different treatments. **h** Schematic illustrations of tumors in different groups of tumor-bearing nude mice after 14 days

The original article has been corrected.

