# Supplementary material for: N-doped carbon dots for dual-modality NIR fluorescence imaging and photothermal therapy
Source: J Nanobiotechnology. 2025 Jul 15;23:513. doi: 10.1186/s12951-025-03497-6 (PMC12261557; doi:10.1186/s12951-025-03497-6)
Supplement: Supplementary file 2 — Supplementary Material 2 [file 12951_2025_3497_MOESM2_ESM.docx]

**Supporting information**

**N-Doped Carbon Dots for Dual-Modality NIR Fluorescence Imaging and Photothermal Therapy**

**Hui-Xian Shi ^1*^, Xuan Qu ^1^, Tong-Tong Zhao ^1^, Zhong-Fu An ^2^,** **Chuan-Yi Zhang^3^, Hong-Liang Wang ^3,4 *^**

^1^ Shanxi Key Laboratory of Biomedical Metal Materials, College of Materials Science and Engineering, Taiyuan University of Technology, Taiyuan 030024, China, E-mail: [shihuixian@tyut.edu.cn](mailto:shihuixian@tyut.edu.cn)

^2^ Key Laboratory of Flexible Electronics (KLOFE) & Institute of Advanced Materials (IAM), Nanjing Tech University, 30 South Puzhu Road, Nanjing 211816, China

^3^ Department of Nuclear Medicine, First Hospital of Shanxi Medical University, Taiyuan, Shanxi, 030001, China.

^4^ Shanxi Key Laboratory of Molecular Imaging & Collaborative Innovation Center for Molecular Imaging of Precision Medicine, Shanxi Medical University, Taiyuan, Shanxi, 030001, China.

*^*^* Corresponding Author: E-mail [shihuixian@tuyt.edu.cn](mailto:shihuixian@tuyt.edu.cn), [hongliang0812@163.com](mailto:hongliang0812@163.com)

**1. Experimental Section.**

**1.1 Materials**

Citric acid (CA) was purchased from Tianjin Guangfu Technology Development Co., Ltd., Sodium hydroxide (NaOH) was obtained from Aladdin Bio-Chem Technology Co., Ltd. (Shanghai, China). Biuret and N, N'-dimethylformamide (DMF) were acquired from Shanghai Macklin Biochemical Co., Ltd., (Shanghai, China). The culture medium (DMEM), EDTA-trypsin, and penicillin/streptomycin were sourced from Gibco BRL (MD, USA). Fetal bovine serum (FBS) was procured from Every green (China). Dimethyl sulfoxide (DMSO) was acquired from Tianjin Damao Chemical Reagent Factory (Tianjin, China). The 3-(4,5-dimethylthiazol^-2^-yl)^-2^,5-diphenyltetrazolium bromide (MTT) and phosphate buffer (PBS) were manufactured by Shanghai Biotechnology Development Co. (DCFH-DA, 4',6-diamidino^-2^-phenylindole (DAPI), and Live-Dead Cell Staining Kit (Calcein-AM/PI) were produced from Beyotime Biotechnology Co., Ltd. (China).

**1.2 Characterization**

The lattice patterns and elemental composition of samples were examined with a transmission electron microscope (TEM, Tecnai G20, FEI, USA), which was fitted with an energy-dispersive X-ray spectroscopy (EDS) system. Crystallographic structure and surface composition were characterized through X-ray diffraction (XRD) and X-ray photoelectron spectroscopy (XPS, K-Alpha, Thermo). The UV-vis-NIR absorption properties of the samples were assessed using a UV-vis-NIR spectrophotometer (PerkinELmer, Llantrisant, CF72 8YW, UK). The fluorescence spectra of the samples were characterized using a fluorescence spectrophotometer (FS5-Spectrofluorometer, Edinburgh, FS5, UK). The feasibility of fluorescence imaging in tumor-bearing mice was investigated using the IVIS spectrometer imaging system（IVIS 243 Lumina III; Perki Elmer, Waltham, MA, USA）.

**1.3 Calculation of photothermal conversion efficiency**

The photothermal conversion efficiency was determined following established calculation methods from prior literature [11], with detailed procedures outlined below: A infrared thermal imager (A308, Guide) was used to record the temperature changes of the N-CDs solution. To evaluate the photothermal conversion efficiency, N-CDs aqueous solution was continuously illuminated by the 808/1060 nm laser with a power of 1.0 W·cm^-2^ for 10 min. After the irradiation was switched off, the temperature decrease was monitored to determine the rate of heat transfer from the system. The photothermal conversion efficiency, η, was calculated according to equation (1-1):

$ƞ=\frac{hs\left( T_{max}-T_{surr} \right)-Q_{pla}}{I\left( 1-{10}^{-A} \right)}\times100\%$ (1-1)

where h is the heat transfer coefficient, S represents the surface area of the container, T_max_ is the equilibrium temperature, T_Surr_ is the room temperature, Q_pla_ is the heat dissipation caused by light absorption in the 96-well microplate, I is the laser power, and A is the absorbance of N-CDs solution at 808/1060 nm. The value of hS was derived according to equation (1-2):

$\tau_{s}=\frac{m_{D}C_{D}}{һS}$ (1-2)

where τ_s_ is the sample system time constant, m_D_ and C_D_ are the mass (0.2 g) and heat capacity (4.2 J/g) of deionized water, respectively.

**1.4 Cell Experiment**

Cell culture: Prostate cancer cells from humans, specifically the DU145 cell line, were grown in Gibco's DMEM medium supplemented with 10% fetal bovine serum (FBS), along with 1% penicillin and streptomycin. The cells were maintained at 37 °C in an environment with 5% carbon dioxide (CO_2_) and 95% atmospheric air.

**1.5 Animal experiment**

Animal Tumor Model: The animal experiments were conducted in accordance with the guidelines for the care and use of laboratory animals of Taiyuan University of Technology and they were approved by the Animal Ethics Committee of the International Animal Welfare Standards (tyut202205001). Male nude mice weight approximately 20 g were obtained from Shanxi Medical University. The right side was subcutaneously inoculated with a DU145 cell suspension (2×10^6^ cells dispersed in 100 µL). once the tumor volume reached about 100 mm^3^, animal studies were performed.

**2. Supporting figures**


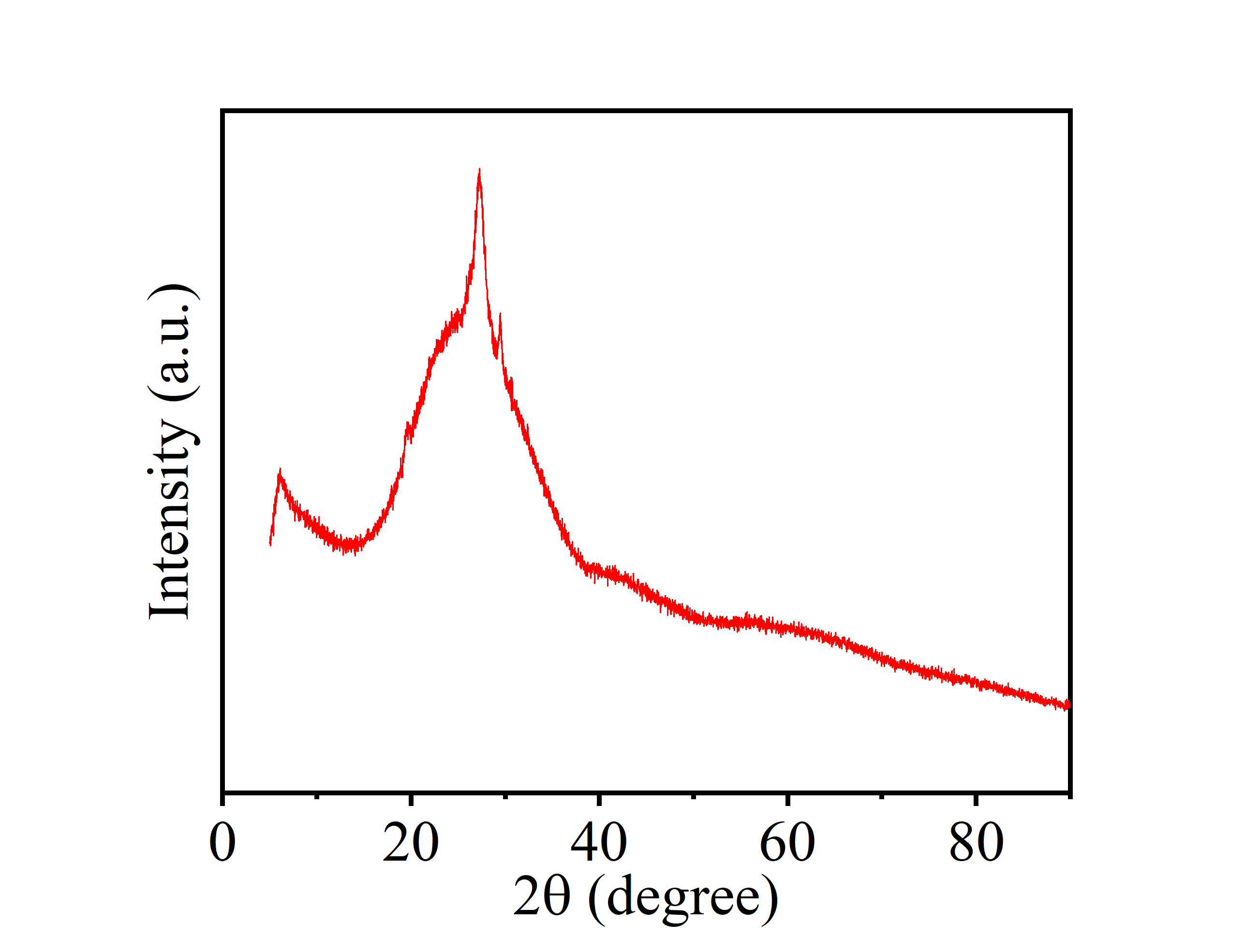


**Fig. S1.** XRD image of N-CDs


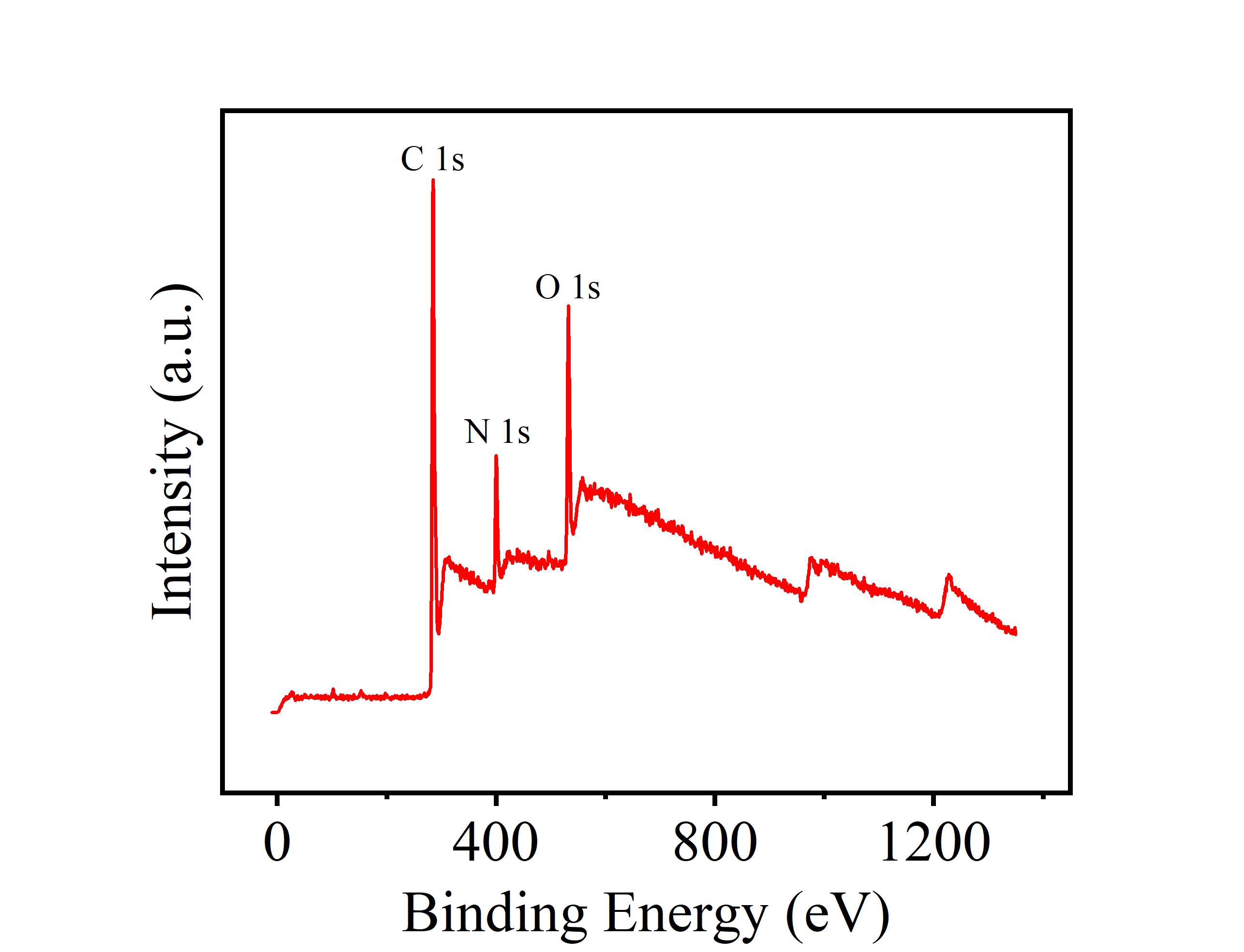


**Fig. S2** XPS wide-scan spectrum of N-CDs

**Table. S1** The ratio of elements

| Name | Peak BE | FWHM (eV) | Area（p）CPS (eV) | Atomic（%） |
| --- | --- | --- | --- | --- |
| N1s | 400.32 | 3.90 | 140521.97 | 11.23 |
| C1s | 284.8 | 4.31 | 602476.07 | 74.66 |
| O1s | 532.25 | 4.39 | 275137.04 | 14.11 |


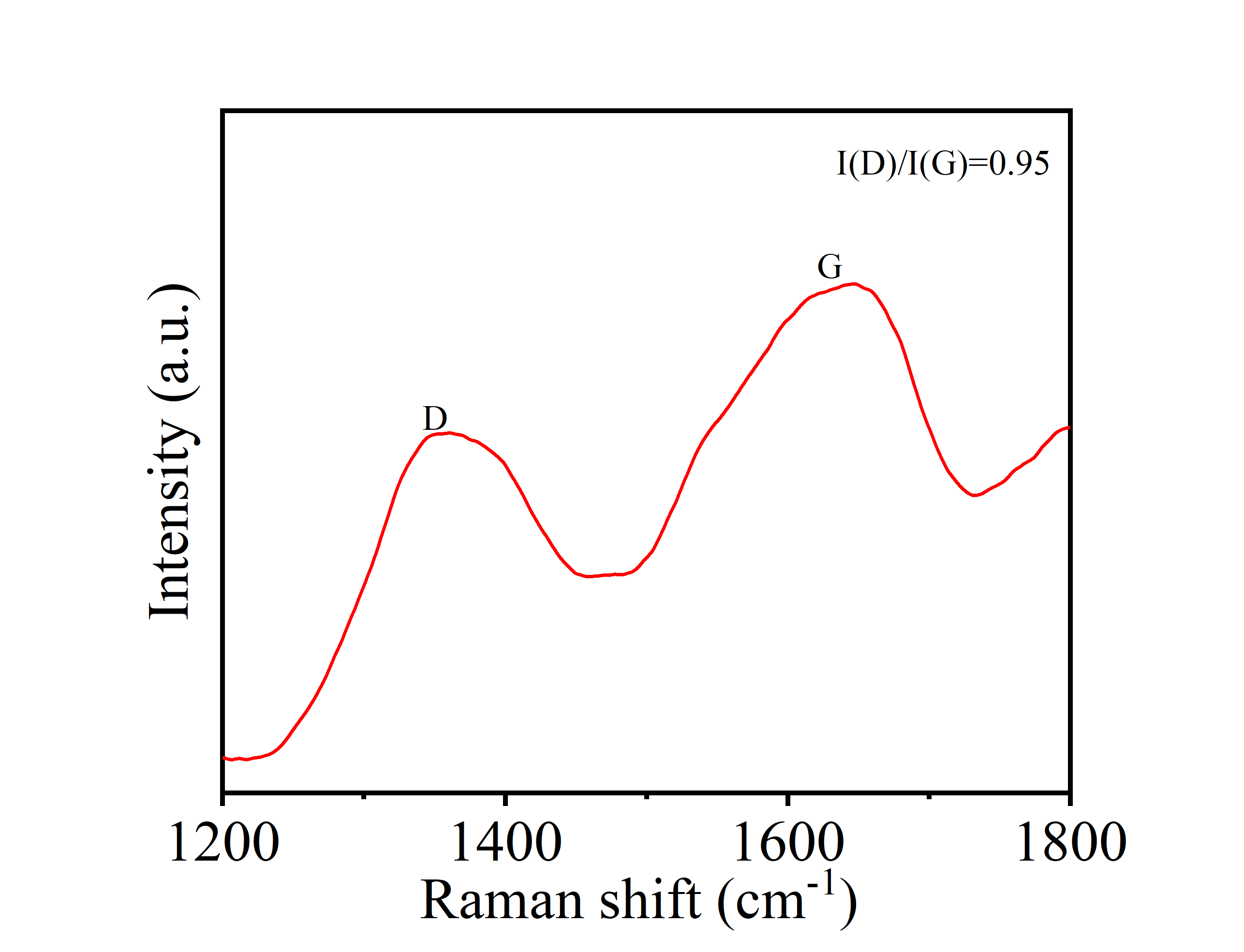


**Fig. S3** Raman spectra of N-CDs (Excitation at 325 nm)


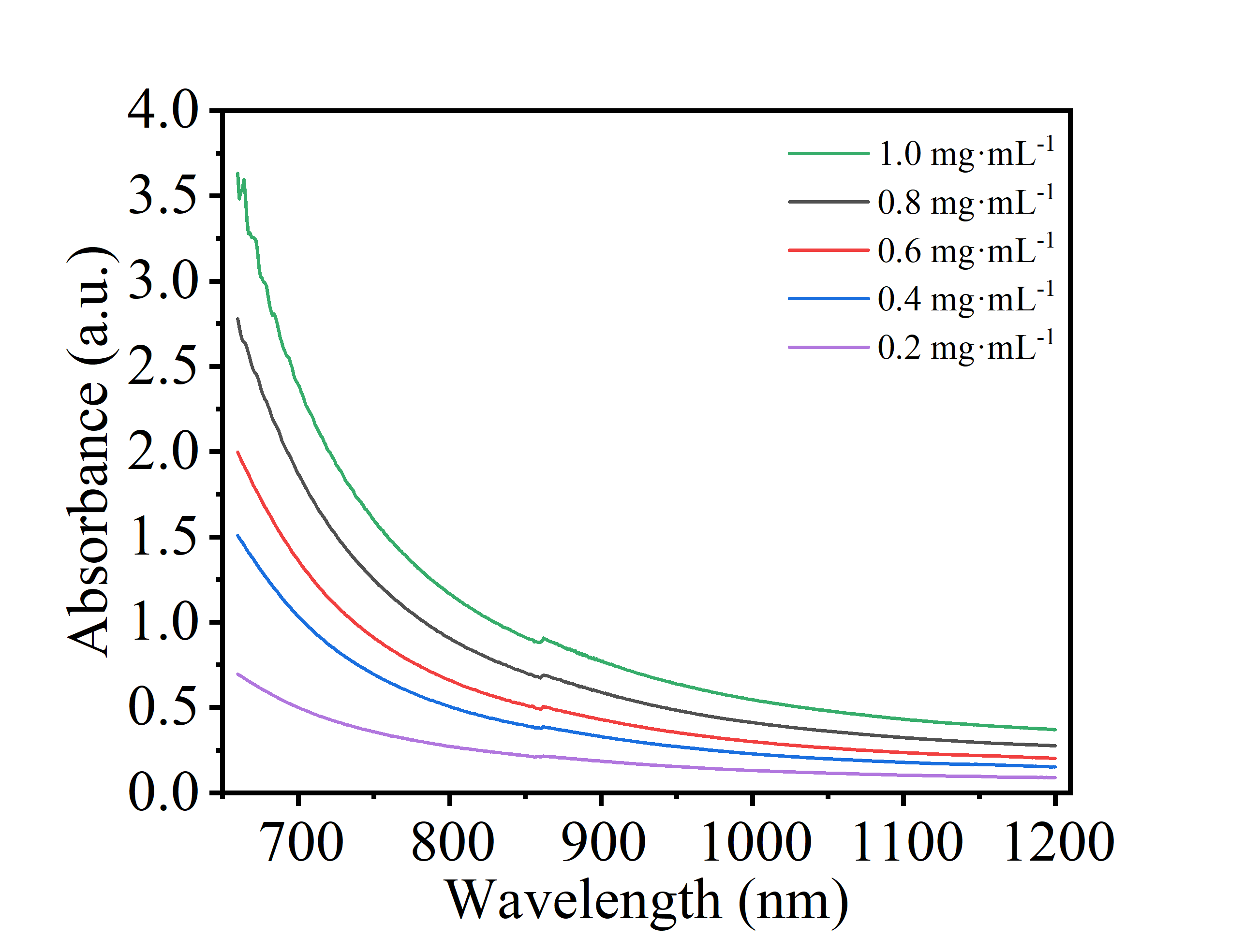


**Fig. S4** UV-vis-NIR absorption spectra of solutions with different concentrations of N-CDs


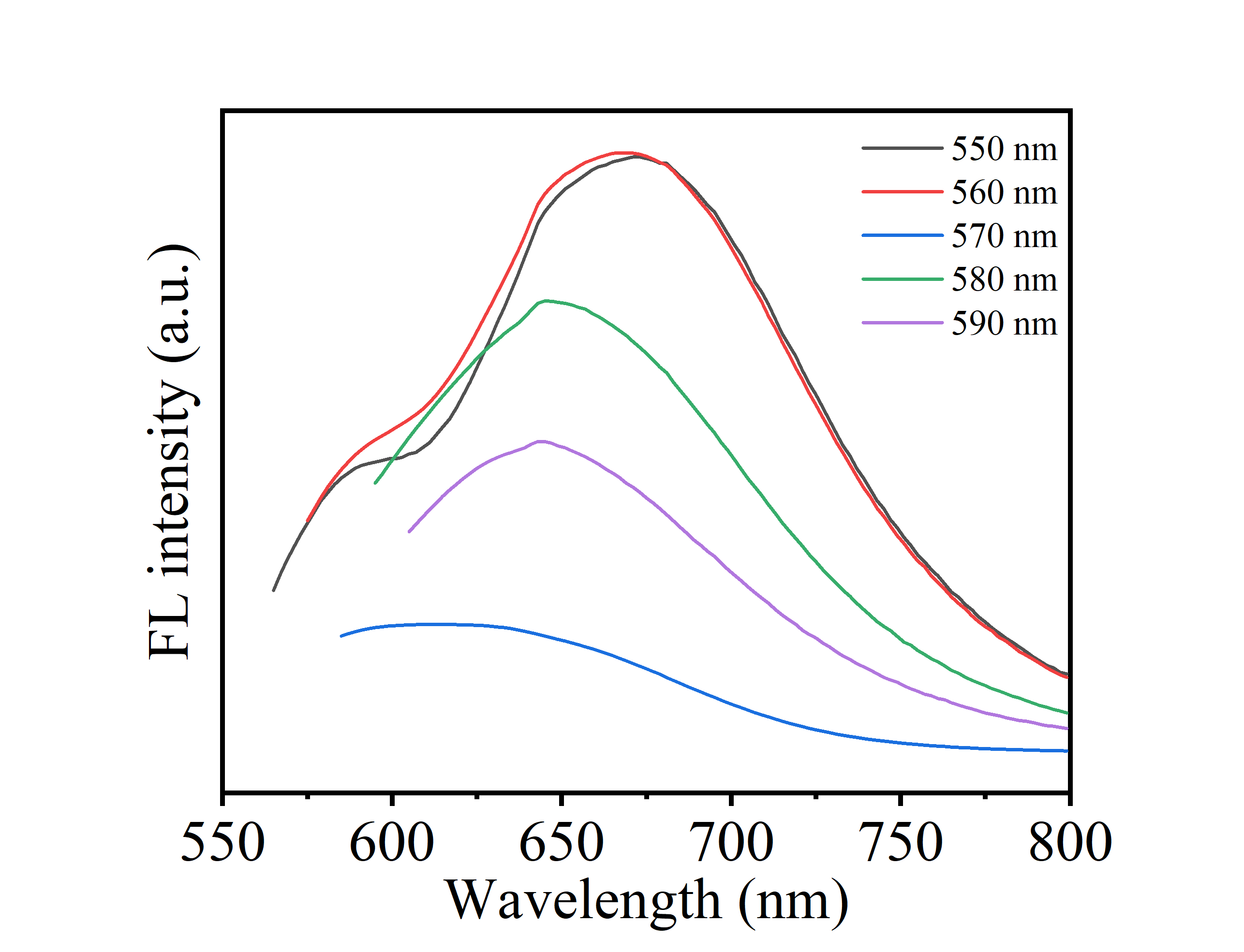


**Fig. S5** Fluorescence spectra of N- CDs under excitation at different wavelengths


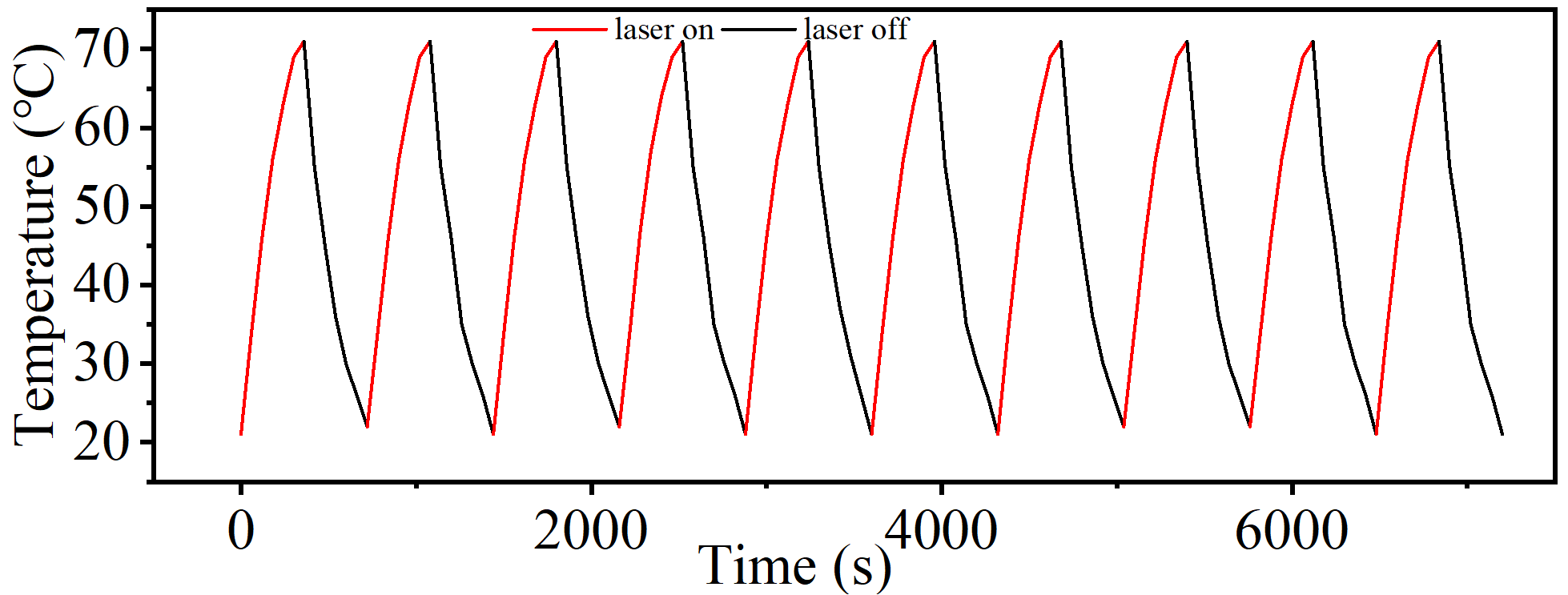


**Fig. S6** Photothermal cycle curve of N-CDs aqueous solution (10 cycles at 808 nm, 1.0 W·cm^–2^)

**Fig. S7** Zeta potential of N-CDs in different media


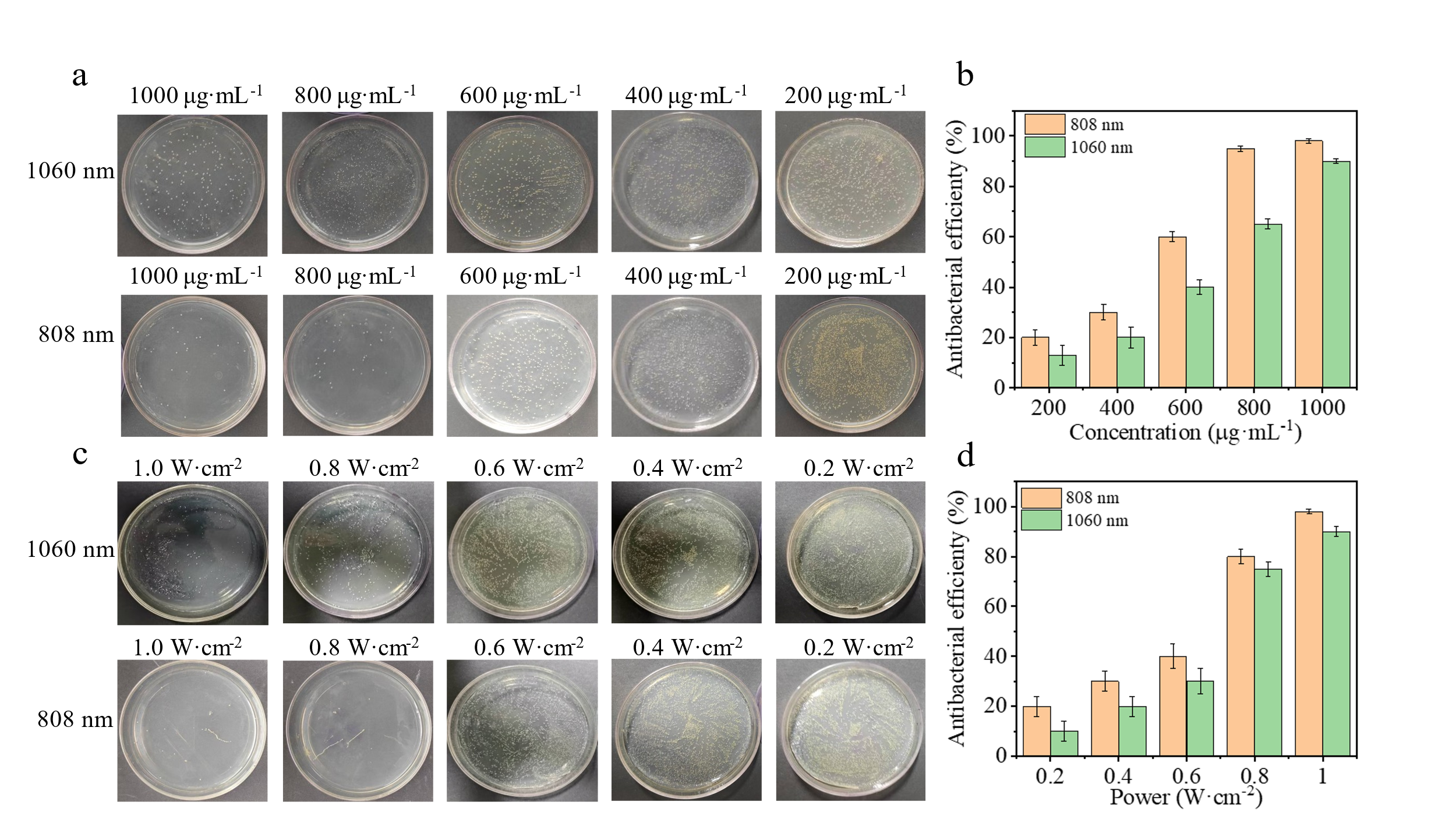


**Fig. S8** (a) Photographs and (b) antibacterial rates of N-CDs solutions with different concentrations assessed by the flat plate spreading method; (c) Photographs and (d) antibacterial rates of N-CDs solutions with different laser powers assessed by the flat plate spreading method.


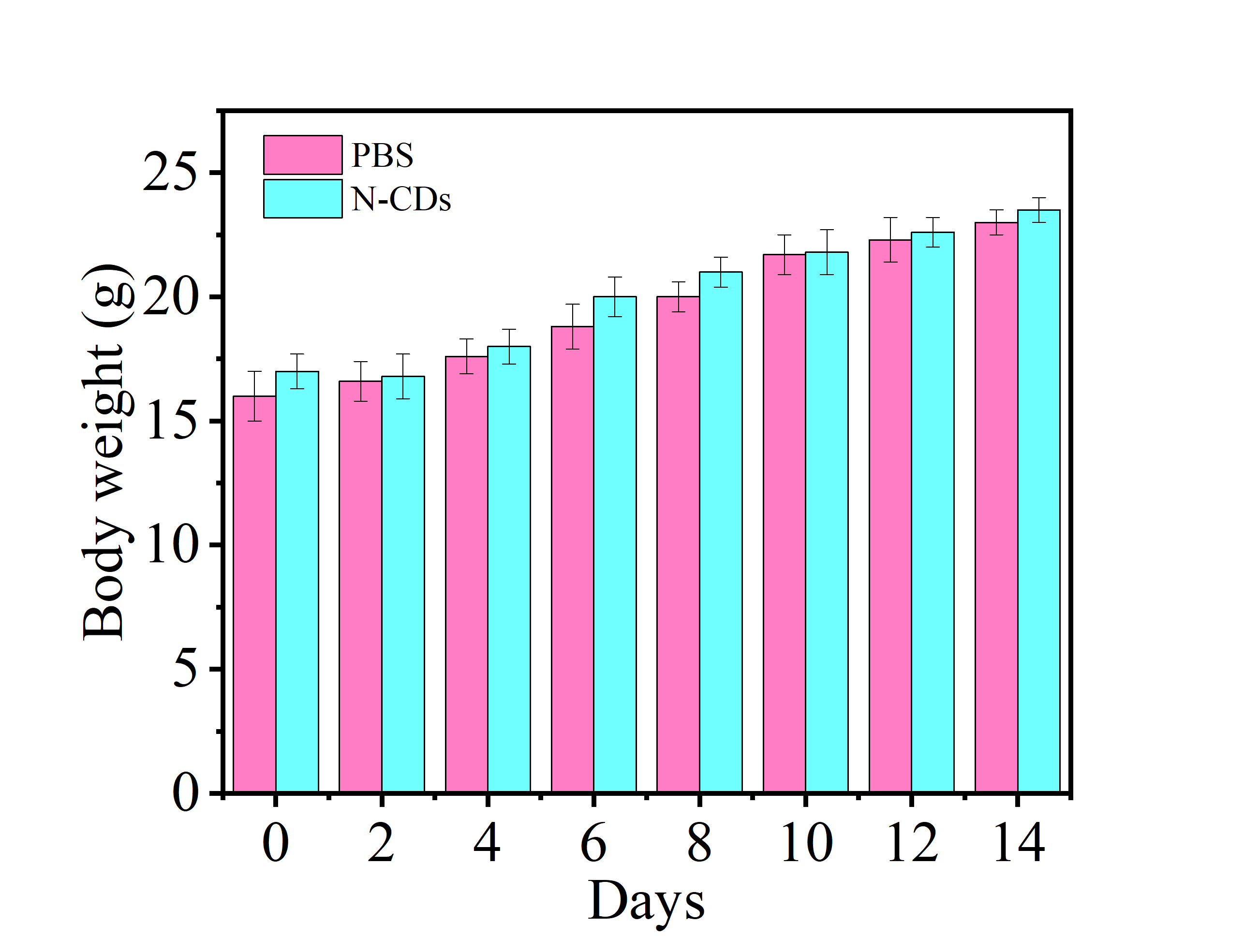


**Fig. S9** Body weight changes in healthy mice within 14 days after injection with N-CDs solution and PBS solution.


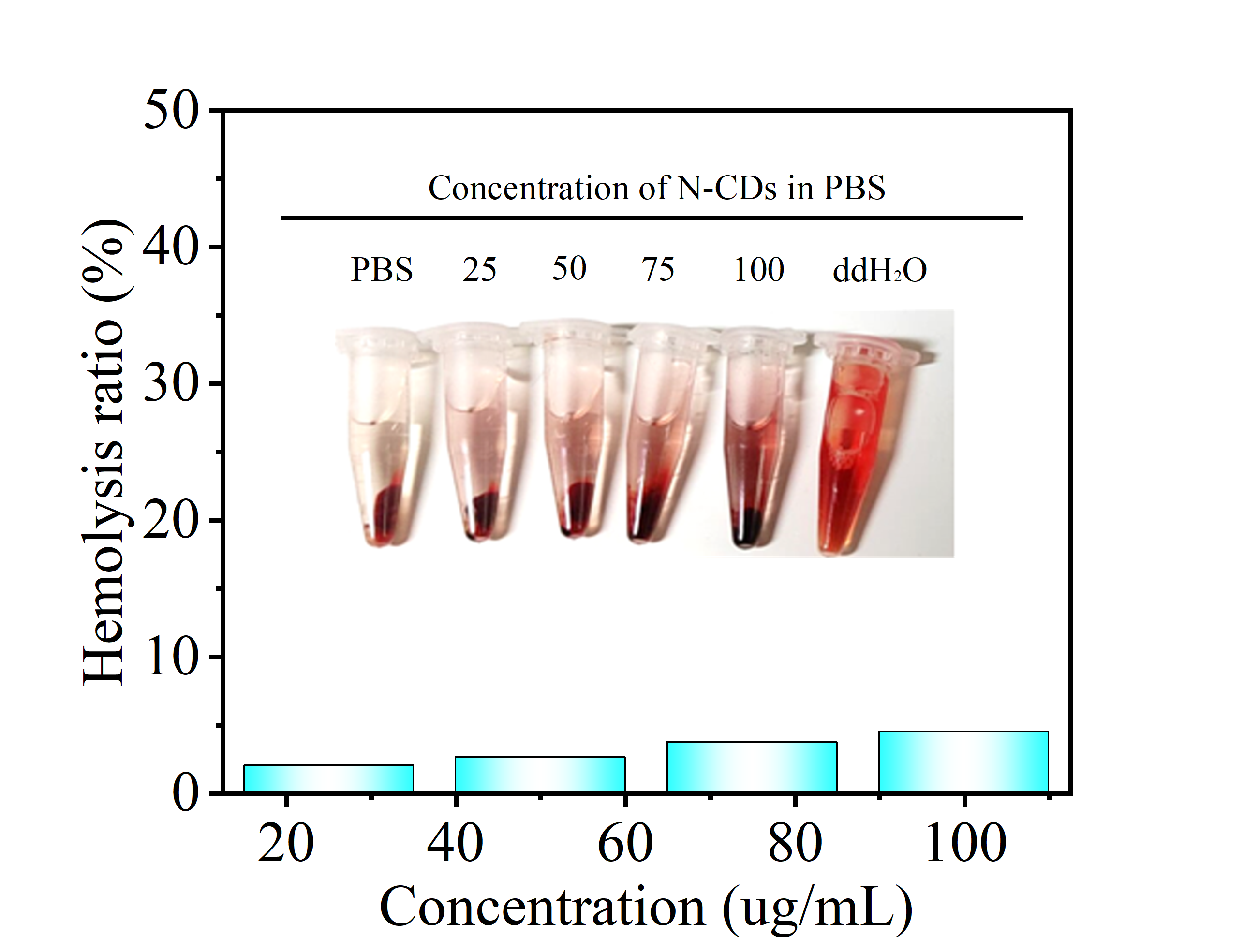


**Fig. S10** Hemolysis of mouse blood caused by N-CDs

**Table. S2** Fluorescence intensity values at different time points within the specific tumor

| Time (h) | Control/FL intensity (a.u.) | N-CDs/FL intensity (a.u.) | The residual amount of N-CDs (μg) | The metabolic rate of N-CDs (%) |
| --- | --- | --- | --- | --- |
| 1 | 9.137E6 | 9.092E7 | 10 | 0 |
| 2 | 7.231E6 | 6.035E7 | 6.64 | 33.6 |
| 4 | 5.020E6 | 4.728E7 | 5.20 | 48.0 |
| 8 | 4.510E6 | 3.661E7 | 4.03 | 59.7 |
| 24 | 4.699E6 | 1.39E7 | 1.53 | 84.7 |


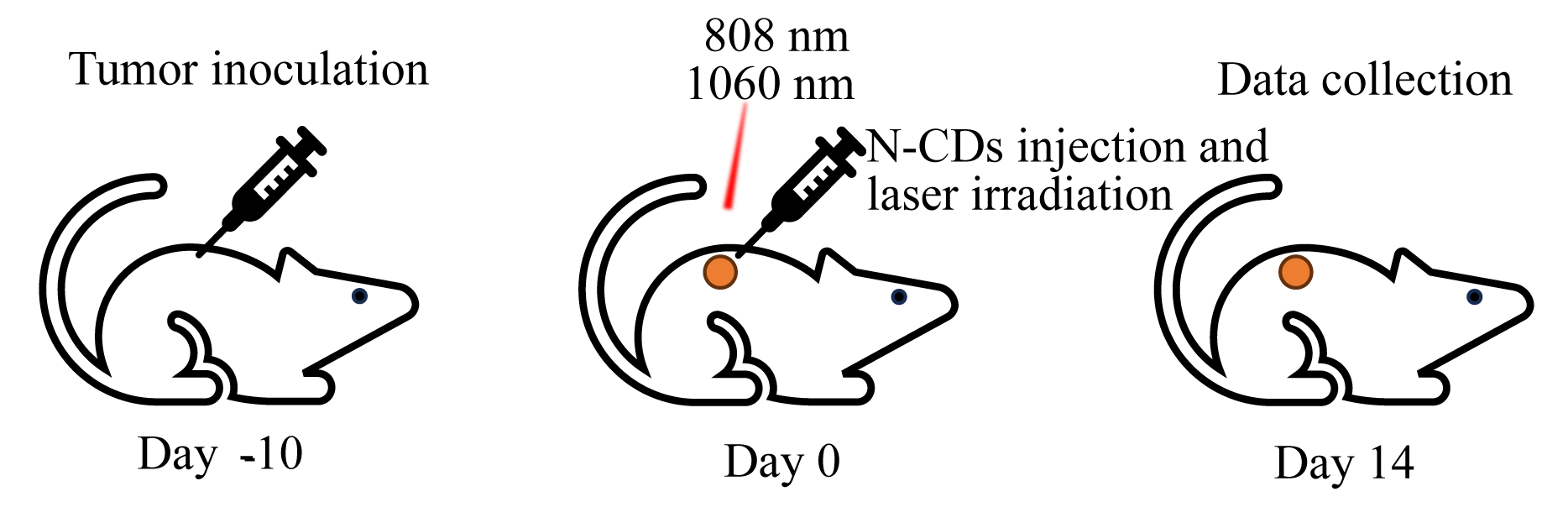


**Fig. S11** Schematic illustration of the PTT process in tumor-bearing nude mice


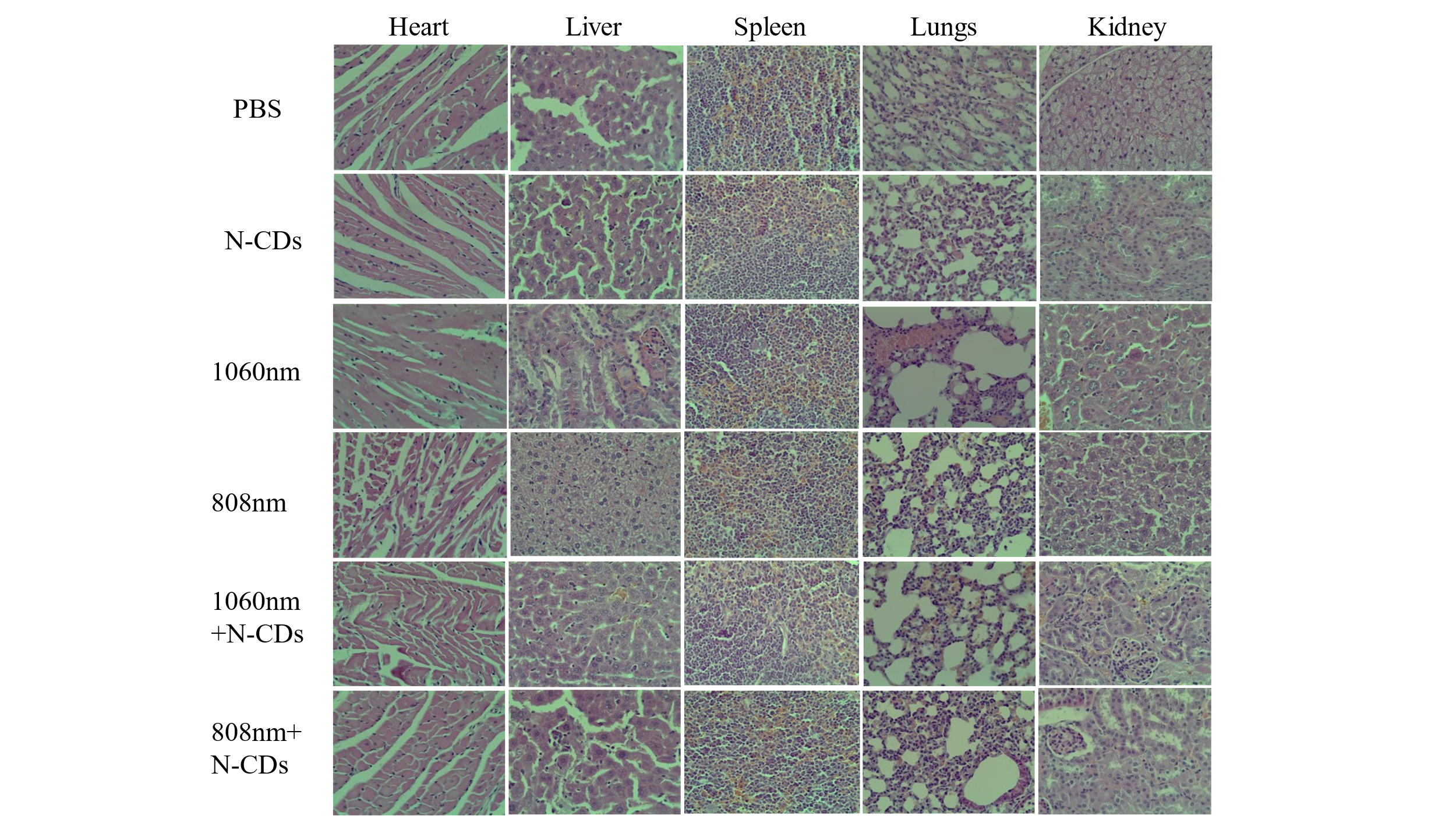


**Fig. S12** H&E staining of the major organs (heart, liver, spleen, lung, kidney) of DU145 tumor-bearing nude mice after 14 days of treatment.


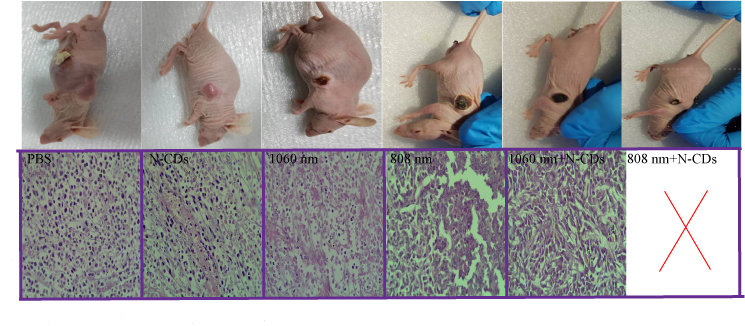


**Fig. S13** Photographs of tumor-bearing nude mice on the 14th day and H&E stained tumor slices from each group
